# Perfusion-based ex vivo culture of frozen ovarian cancer tissues with preserved tumor microenvironment

**DOI:** 10.1038/s41698-025-00941-6

**Published:** 2025-05-23

**Authors:** Monica De Luise, Ivana Kurelac, Sara Coluccelli, Antonio De Leo, Ewelina M. Bartoszek, Maria Iorio, Marco Grillini, Camelia Alexandra Coadă, Dario de Biase, Lorena Marchio, Mónica Núñez López, Natalie Rimmer, Anna Myriam Perrone, Pierandrea De Iaco, Anna Maria Porcelli, Viola Heinzelmann, Ivan Martin, Francis Jacob, Manuele Giuseppe Muraro, Giuseppe Gasparre

**Affiliations:** 1 Department of Medical and Surgical Sciences (DIMEC), University of Bologna, Bologna, Italy; 2 Centre for Applied Biomedical Research, University of Bologna, Bologna, Italy; 3 IRCCS Azienda Ospedaliero-Universitaria di Bologna, Bologna, Italy; 4 Solid Tumor Molecular Pathology Laboratory, IRCCS Azienda Ospedaliero-Universitaria di Bologna, Bologna, Italy; 5 Microscopy Core Facility, Department of Biomedicine, University of Basel, Basel, Switzerland; 6 Tissue Engineering, Department of Biomedicine, University of Basel and University Hospital of Basel, Basel, Switzerland; 7 Ovarian Cancer Research, Department of Biomedicine, University of Basel and University Hospital of Basel, Basel, Switzerland; 8 Pathology Unit, IRCCS Azienda Ospedaliero-Universitaria di Bologna, Bologna, Italy; 9 Department of Pharmacy and Biotechnology (FABIT), University of Bologna, Bologna, Italy; 10 Division of Gynecologic Oncology, IRCCS Azienda Ospedaliero-Universitaria di Bologna, Bologna, Italy; 11 Department of Pharmacy and Biotechnology (FABIT) and Centre for Applied Biomedical Research (CRBA), University of Bologna, Bologna, Italy; 12 Department of Biomedical Engineering, University of Basel, Basel, Switzerland; 13 Centro Studi E Ricerca Sulle Neoplasie Ginecologiche (CSR), University of Bologna, Bologna, Italy

**Keywords:** Ovarian cancer, Translational research, Cancer models

## Abstract

Ovarian cancer (OC) poses significant treatment challenges due to late-stage diagnosis and a complex tumor microenvironment contributing to therapy resistance. We optimized a U-CUP perfusion-based bioreactor method to culture patient-derived primary and metastatic OC specimens, demonstrating that perfusion better preserves cancer cell viability and proliferation, both when fresh and slow-frozen tissues were used. Perfused cultures maintained key microenvironment components, including cancer-associated fibroblasts, endothelial and immune cells. Genetic analysis confirmed the retention in culture of tumor-specific driver mutations. We hence challenged ad hoc generated cisplatin-sensitive and resistant OC cells with cisplatin during growth in U-CUP, validating our system for the testing of drug response. Finally, treatment of slow-frozen OC tissues with carboplatin/paclitaxel revealed different degrees of response to treatment, as indicated by variations in tumor necrosis and number of residual PAX8^+^ cells, providing the bases for the prompt evaluation of OC standard chemotherapy efficacy in our ex vivo system.

## Introduction

Ovarian cancer (OC) remains a significant public health challenge worldwide, with an estimated 314,000 new cases and 207,000 deaths annually^1^. Due to its asymptomatic nature in the early stages, the disease is often diagnosed at an advanced stage, contributing to its high mortality rate. As a result, OC is the eighth most common cancer among women and ranks fifth in cancer-related deaths^1^. The overall 5-year survival rate for patients diagnosed at advanced stages (FIGO III-IV) is between 30–40%^2^. Current therapeutic strategies include primary or interval cytoreductive surgery, the latter being preceded by at least 3–4 cycles of neoadjuvant chemotherapy^3^. Despite these efforts, high recurrence rates and resistance to standard treatments like platinum-based compounds and taxane remain major obstacles^4^. The addition of anti-angiogenic inhibitors like Bevacizumab has shown promise in primary treatment, but it is not typically used in maintenance therapy, which currently envisions the administration of PARP inhibitors for patients with *BRCA1/2* mutations and homologous recombination deficiency (HRD)^3^. However, even with these advances, the development of treatment resistance remains a significant challenge, largely due to the high degree of heterogeneity of the tumor microenvironment (TME)^5,6^. The complex TME, comprising cancer-associated fibroblasts that support tumor growth and immune suppression, tumor-associated macrophages promoting inflammation and immune evasion, endothelial cells facilitating angiogenesis, and immune cells influencing tumor progression, plays a crucial role in fostering therapy resistance^7^. Therefore, understanding the TME is essential for identifying new therapeutic targets and overcoming treatment resistance.

Traditional in vitro models, such as 2D cultures and 3D spheroids, are limited in their ability to capture the heterogeneity of the TME^8,9^. Although co-culture systems may help dissect molecular mechanisms, they require labor-intensive processes and still fall short of replicating patient tumor tissue complexity^10^. While patient-derived organoids (PDOs) reflect cancer heterogeneity more effectively, they remain limited in recreating the full complexity of the human TME, particularly the lack of stromal cells and the dynamic perfusion of nutrients and waste, leading to inconsistencies in clinical translation^11–13^. Furthermore, murine patient-derived xenograft models (PDXs) require humanized systems to incorporate immune cell components, making them overly time-consuming and costly for high-throughput drug screening^14^.

A promising innovation that addresses these limitations is the use of perfusion-based bioreactor technology, which has demonstrated an ability to generate tissue constructs with biological and structural characteristics closely resembling original tissues^15–18^. Unlike static culture systems, which have limitations in replicating the dynamic aspects of the TME, perfusion-based models allow for continuous nutrient flow and waste removal, more effectively preserving tissue architecture and the microenvironment ex vivo^19–21^. Previous studies from our group have shown the suitability of this technology for tumor engineering purposes, further validating its potential in ovarian cancer modeling^22–24^.

Building on this previous work, we developed ex vivo cultures of patient-derived OC tissues including tubo-ovarian high grade serous carcinoma (HGSC) samples and one low-grade serous carcinoma (LGSC) sample, using the U-CUP bioreactor. We assessed cancer cell viability and TME integrity under ex vivo culture conditions in both Fresh and biobanked Slow-Frozen (SF) specimens. Furthermore, we evaluated the efficacy of standard chemotherapy (SCT) regimen during culture. We demonstrate for the first time that the U-CUP perfusion-based culture maintains ovarian cancer tissue viability and TME architecture and is, thus, a valuable tool for studying not only standard chemotherapy but also for testing novel pharmacological strategies.

## Results

### Perfusion enhances ex vivo OC tissue preservation and proliferation with respect to static culture

Perfusion-based systems have improved the culturing of several cancer types, with optimal preservation of cellular composition, matrix, and tissue architecture in breast and glioblastoma, as compared to static cultures^19,21^. To explore the feasibility of perfused ex vivo cultures of OC, we optimized the U-CUP bioreactor for patient-derived serous OC specimens. Freshly obtained primary tumors and, notably, omental metastases from 5 different patients (Table S1) were cultured for 6 days in perfusion (Pd6) and static (Sd6) conditions in parallel, to compare OC tissue viability, histological morphology, and proliferation. For each approach, three to five tissue chunks were cultured and compared to the corresponding freshly collected sample (d0), which also consisted of three to five tissue chunks derived from the same initial patient tissue, following standardized sectioning and randomization procedures.

**Fig. 1:**
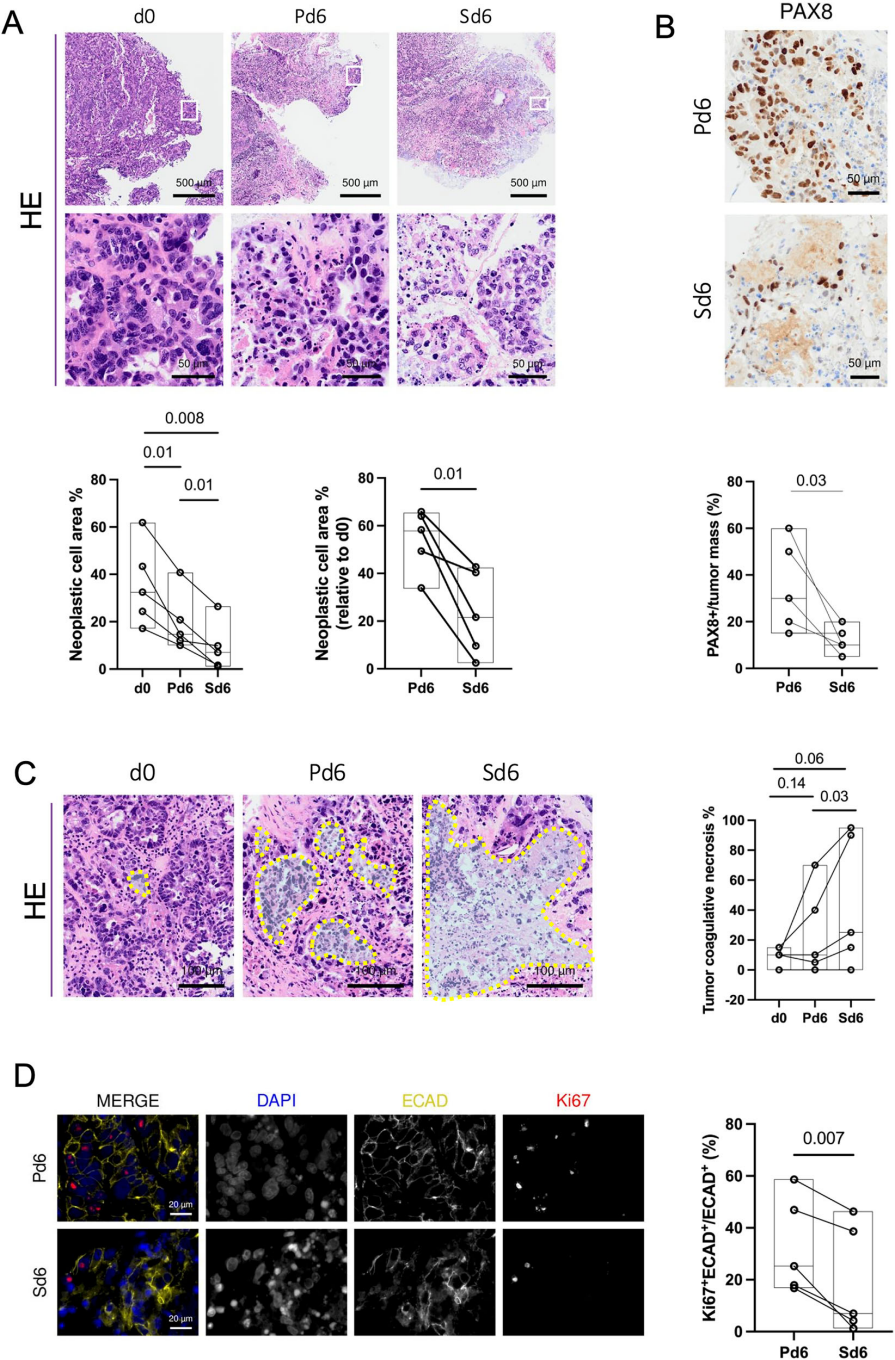
Perfusion enhances ex vivo ovarian cancer (OC) tissue preservation and proliferation with respect to static culture. **A** Representative images of haematoxylin and eosin (HE) staining of uncultured HGSC tissues and the LGSC OC-3 tissue (d0), and the corresponding 6-day culture in U-CUP perfused (Pd6) or static conditions (Sd6). White squares indicate the insets of the areas shown at higher magnification. Line plots represent the percentage of neoplastic cell area, quantified in each uncultured HGSC tissue and in the LGSC (OC-3) tissue (d0), paired to corresponding perfused (Pd6) and static (Sd6) 6-day cultures. The floating bar overlay indicates minimum to maximum, and the median value (black line) for each dataset. QuPath was used for quantification. Paired t-tests were applied for dataset comparison (*n* = 5). **B** Representative images of PAX8 immunohistochemistry in HGSC specimens and in the LGSC (OC-3) specimen cultured in perfused (Pd6) and static (Sd6) conditions. Line plot indicates the percentage of PAX8^+^ cells in the tumor mass quantified in paired tissues. The floating bar overlay indicates minimum to maximum, and the median value (black line) for each dataset. Quantification was performed by a trained pathologist. One-tailed paired t-test was applied for dataset comparison (*n* = 5). **C** Representative HE images of necrotic areas (dashed lines) in uncultured HGSC tissues and the LGSC (OC-3) tissue (d0) and the corresponding 6-day culture in U-CUP perfused (Pd6) or static (Sd6) conditions. Line plot indicates the percentage of necrosis in the tumor mass quantified in paired tissues. The floating bar overlay indicates minimum to maximum, and the median value (black line) for each dataset. Quantification was performed by a trained pathologist. The LGSC sample (OC-3) (flat line) is plotted for data transparency without being included in the statistical analysis due to the absence of tumor necrosis in these tumors. One-tailed paired t-tests were applied for dataset comparison of HGSC (*n* = 4). **D** Representative IF images of HGSC specimens and the LGSC (OC-3) specimen cultured in perfused (Pd6) and static (Sd6) conditions. Nuclei (DAPI, blue), epithelial cells (ECAD, dark yellow), and the proliferative marker Ki67 (Ki67, red) are shown in MERGE. The single fluorescence channels are shown separately in black/white images. Line plot represents the percentage of Ki67^+^ECAD^+^ cells normalized to all ECAD^+^ cells present in the neoplastic cell area. The floating bar overlay indicates minimum to maximum, and the median value (black line) for each dataset. QuPath was used for quantification. Paired t-test was applied for dataset comparison (*n* = 5). For all panels, p-values are shown within the graphs.

As an indicator of tumor preservation, we first assessed the percentage of neoplastic cell area in the specimens using haematoxylin and eosin (HE) staining, defined as the area predominantly occupied by cancer cells, excluding tumor stroma and adjacent normal tissue (Fig. S1A). Despite an overall decrease in the neoplastic cell area compared to d0 in both culture types (Pd6 median 58.30%; Sd6 median 21.62%), better maintenance was observed under perfusion (*p* = 0.01) (Fig. 1A).

To ensure that the neoplastic area contained viable cancer cells, we evaluated the positivity for Paired box gene 8 (PAX8), a well-established epithelial marker strongly expressed in serous OC^25^. The percentage of PAX8^+^ cells in the tumor mass was higher in perfused culture, when compared to the static counterpart (*p* = 0.03) (Fig. 1B), highlighting the advantages of perfusion in maintaining tumor histological morphology and antigenicity. We ruled out nuclear phenotypic alterations by measuring the nuclear size of PAX8^+^ cells. The analysis revealed comparable nuclear sizes across all conditions, including static and perfused samples, relative to uncultured controls (d0) (Fig. S2; Table S7).

Moreover, given that insufficient nutrient and oxygen supply can lead to cell death, more likely to occur during in vitro culturing, we evaluated the extent of coagulative necrosis in the cultured tissues. Our attempt was to validate that bioreactor-driven perfusion may help mitigate this issue by ensuring a more efficient supply of medium to the tissue, thereby improving survival and reducing tumor cell death. At day 6, both perfused and static cultures showed a higher median necrosis compared to Fresh (d0) specimens (d0, median 10%; Pd6 median 25%; Sd6 median 57%) (Fig.1C), with evident nuclear changes such as pyknosis, karyorrhexis, and karyolysis. However, perfused chunks displayed less tissue coagulative necrosis than Sd6 tissues (Pd6 median 25%; Sd6 median 57%; *p* = 0.03) (Fig. 1C). The only exception was a LGSC case, which displayed no necrosis in all tested condition (Fig. 1C), consistent with the fact that LGSC is characterized by the absence of necrosis^26^. These results suggest that perfusion is indeed useful to partly mitigate the detrimental effects on cell viability triggered in vitro by facilitating nutrient supply to the inner portions of the tissue chunks.

Finally, to confirm that perfused conditions more effectively sustain OC cell division, we evaluated the mitotic index in epithelial cancerous cells using Ki67 and the epithelial marker E-cadherin (ECAD) by immunofluorescence (IF) in cultured OC tissues, revealing significant differences in cell proliferation and epithelial cell morphology between the two conditions (Fig. 1D). In Pd6 samples, DAPI staining indicated well-preserved nuclei, and ECAD staining showed continuous epithelial cell junctions, reflecting a maintained tissue architecture^27^. In contrast, Sd6 samples displayed occasional intact nuclei and fragmented ECAD staining, indicative of disrupted epithelial integrity^28^. Quantification of Ki67^+^ cells revealed a higher proliferative index in Pd6 samples, compared to Sd6 counterparts (Pd6 median 22.8%; Sd6 median 7.0%; *p* = 0.007), underscoring the superiority of the perfusion-based culturing system in supporting epithelial cancer cell proliferation and overall preserving the structural characteristics of OC tissues compared to static culture.

### Cell viability and proliferation are preserved in perfused, slow-frozen OC culture

The availability of fresh tissue from patients represents a significant challenge in translational research, often limited by the accessibility of surgical facilities and equipped laboratories. This underscores the pressing demand for methodologies capable of leveraging patient sample reservoirs within biobanks. Our previous work demonstrated that removing cryoprotective agents under perfused flow in the U-CUP bioreactor increases the viability of mesenchymal stromal cell-based SF 3D tissue constructs more effectively than conventional methods^29^. In this study, we extended the validation of U-CUP efficacy in maintaining the integrity of SF ex vivo OC specimens.

**Fig. 2:**
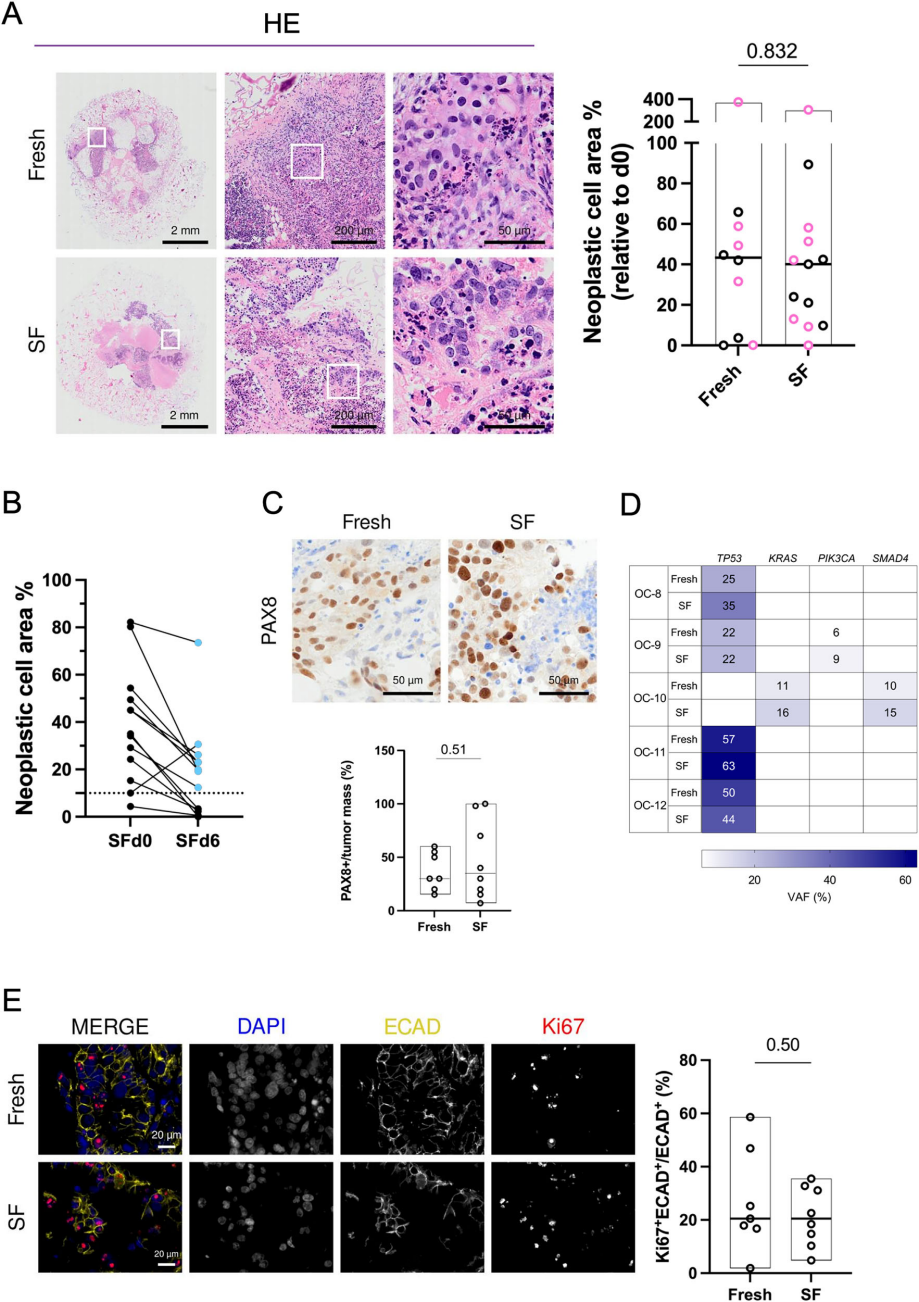
Cell viability and proliferation are preserved in perfused, slow-frozen ovarian cancer (OC) culture. **A** Representative images of haematoxylin and eosin (HE) staining of perfused cultures derived from Fresh or Slow-Frozen (SF) tissues. White squares indicate the insets of the areas shown at higher magnification. The floating bar graph shows the neoplastic cell area percentage in perfused cultures derived from HGSC and the LGSC (OC-3) Fresh (*n* = 10) or HGSC SF (*n* = 13) specimens, with respect to the uncultured tissue (d0). Black and pink circles indicate primary tumors and metastases, respectively. Minimum, maximum, and median values (black line) are indicated for each dataset. QuPath was used for quantification. **B** Line plot represents the percentage of neoplastic cell area in SF HGSC chunks upon thawing (SFd0) and after 6-day culture in U-CUP (SFd6), with respect to the specimen’s area. The dotted line separates the samples with residual neoplastic cell area ≥10% (cyan dots) (*n* = 13). **C** Representative images of PAX8 immunohistochemistry in perfused cultures derived from Fresh or SF tissues. The floating bar graph shows the percentage of PAX8^+^ cells in the tumor mass. Minimum, maximum, and median values (black line) are indicated for each dataset (HGSC and the LGSC OC-3 Fresh *n* = 7, HGSC SF *n* = 8). Quantification was performed by a trained pathologist. **D** Variant allele frequency (VAF) percentage of TP53, KRAS, PIK3CA, and SMAD4 mutations in perfused cultures derived from HGSC Fresh or SF tissues. **E** Representative IF images of perfused cultures derived from Fresh or SF tissues. Nuclei (DAPI, blue), epithelial cells (ECAD, dark yellow), and the proliferative marker Ki67 (Ki67, red) are shown in MERGE. The single fluorescence channels are shown separately in black/white images. The floating bar graph represents the percentage of Ki67^+^ECAD^+^ cells normalized to all ECAD^+^ cells present in the neoplastic cell area (HGSC and the LGSC OC-3 Fresh *n* = 7, HGSC SF n = 8). Minimum to maximum, and the median values (black line) are shown for each dataset. QuPath was used for quantification. For all data presented in the floating bar graphs, unpaired t-tests were applied for comparison and p-values are shown within the graphs.

We first tested whether SF material exhibits comparable performance to Fresh samples when cultured under perfusion. To assess tissue preservation, we evaluated HE sections and measured the percentage of neoplastic cell area in 10 Fresh and 13 SF specimens derived from 17 patients (Table S1). Histopathologic appearance, including cellular morphology, nuclear integrity, and tissue organization, were comparable between both culture conditions (Fig. 2A). Consistent with our comparison (Fig. 1A), we observed an approximate 40% reduction in median neoplastic cell area with respect to d0 for both Fresh and SF settings (Fresh median 43.3%; SF median 40%) (Fig. 2A), suggesting that slow-freezing and thawing marginally induce further damage to the viable cancer tissue. No differences in tissue preservation were observed when either primary or metastatic tissues were cultured (Fig. 2A, Fig. S3). Of note, in a small subset of cases (8.7%), we observed an increase in neoplastic tissue area after 6 days in culture compared to d0. Next, we aimed to define the minimal starting neoplastic cell area within the specimen that may guarantee sufficient viable tissue after 6-day culture to permit informative downstream analyses. Specifically, the neoplastic cell area of SF chunks was evaluated upon thawing (SFd0) and after a 6-day culture in U-CUP (SFd6). Our analysis indicated that to obtain a post-perfusion neoplastic cell area of at least 10%, a value we gauged sufficient for downstream evaluations, a minimum of ∼30% must be present in the thawed SFd0 specimen to ensure a success rate of approximately 80% (Fig. 2B, Fig. S4). Based on these findings, we selected only samples with residual tumor area ≥10% and compared tissue integrity parameters between Fresh and SF cultures, resulting in the analysis of 7 and 8 specimens, respectively. PAX8 staining revealed comparable cancer cell viability between Fresh and SF cultures (Fig. 2C), which was accompanied by only a marginal difference in tumor coagulative necrosis, implying effective tumor preservation under both culture conditions (Fig. S5). These findings were also supported by the analysis of nuclei that confirmed comparable sizes among Fresh and SF perfused samples, excluding the presence of possible phenotypic alterations due to the slow-freezing process (Fig. S2; Table S7).

Finally, we demonstrated that SF cultures preserve cancer cells harboring genetic drivers of the disease by comparing the genetic profile between perfused Fresh and SF tissue. The genes included in the analyses were selected based on clinical guidelines^30^. We sequenced the entire coding region (CDS) of the *TP53* tumor suppressor gene, altered in over 96% high-grade serous OCs^31^, as well as the mutational ‘hot spot’ regions of the *KRAS* oncogene, associated with the LGSC subtype^32^. The analysis was conducted on specimens from 5 patients for which sufficient material was available (Table S1). All patients harbored mutations in at least one of the investigated genes, and all cultures deriving from SF tissue shared the same variants found in freshly perfused specimens (Fig. 2D, Table S2). This concordance in the genetic profiles between different specimens of each patient establishes that slow freezing maintains driver-carrying OC cells in U-CUP culture.

Lastly, IF staining revealed the preservation of ECAD across both SF and Fresh OC specimens. The homogeneous intensity and localization of ECAD at the cell-cell junctions in both sets of samples highlight the efficacy of the slow freezing process in maintaining epithelial characteristics and intercellular adhesion (Fig. 2E). Quantification of Ki67 expression in epithelial cells demonstrated a comparable level of mitotic index between Fresh and SF specimens (Fig. 2E), suggesting OC cells retained proliferative capacity even after slow freezing. These results underscore the potential of SF specimens from biobanks in supporting translational research by retaining key biomarkers essential for studying cancer progression and therapeutic responses.

### Tumor microenvironment components are preserved in perfused, slow-frozen OC culture

Given the crucial role of non-neoplastic cells in influencing therapy response, it is essential that ex vivo preclinical models adequately reflect TME components^33,34^. In this context, we here aimed to demonstrate that our perfusion-based ex vivo culture system not only maintains the TME in freshly perfused OC but also preserves these components after the slow-freezing process.

**Fig. 3:**
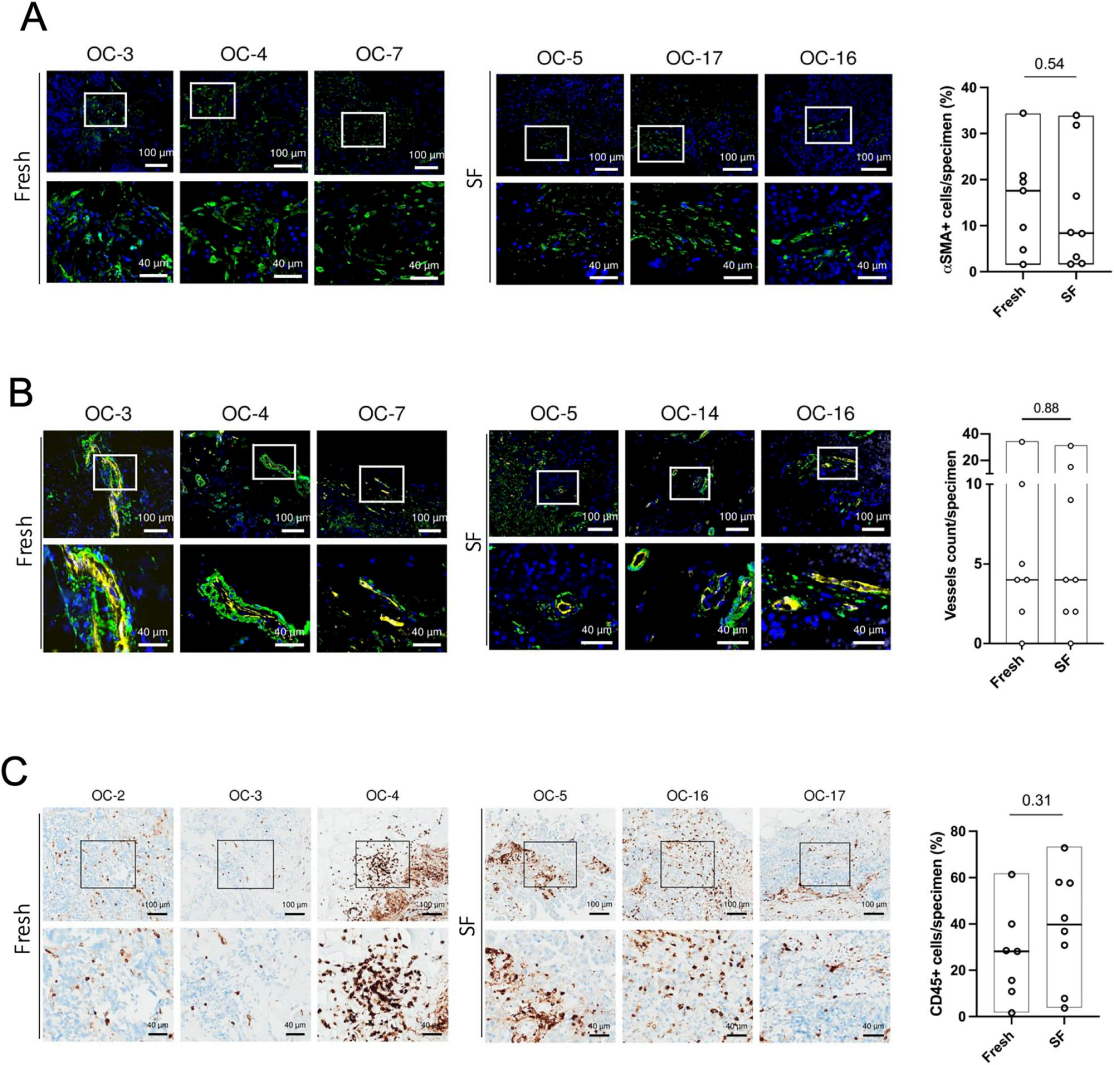
Tumor microenvironment components are preserved in perfused, slow-frozen ovarian cancer (OC) culture. **A** Representative IF images of perfused cultures derived from Fresh HGSC and the LGSC (OC-3) tissues or SF HGSC tissues. Nuclei (blue) and a stromal marker αSMA (green) are shown. The floating bar graph shows the percentage of αSMA^+^ cells in the specimen. **B** Representative IF images of perfused cultures derived from Fresh HGSC and the LGSC (OC-3) tissues or SF HGSC tissues. Nuclei (blue), the pericyte marker αSMA (green), and the endothelial marker CD31 (yellow) are shown in MERGE. White squares indicate the insets of the areas shown at higher magnification. Floating bar graphs represent the number of αSMA^+^CD31^+^ vessels per specimen (vessel count) found in perfused cultures derived from Fresh or SF tissue. **C** Representative images of CD45 immunohistochemistry in perfused cultures derived from Fresh HGSC and the LGSC (OC-3) tissues or SF HGSC tissues. Black squares indicate the insets of the areas shown at higher magnification. The floating bar graph shows the percentage of CD45^+^ cells in the specimen. For all panels, 7 Fresh and 8 SF samples were used, and representative images of cultures derived from 3 different patients are shown. Floating bar graphs indicate minimum, maximum, and median values (black line) for each dataset. QuPath was used for quantification; unpaired t-tests were applied for comparison; and *p*-values are shown within the graphs.

We assessed stromal integrity using alpha smooth muscle actin (αSMA) and podoplanin (D2-40) staining to identify cancer associated fibroblasts (CAFs) and CD31 for endothelial cells. Given that αSMA is also a marker for pericytes coating mature vasculature^35^, we focused our analysis on CD31^+^ structures that were associated with αSMA^+^ cells. Additionally, potential immune infiltrates were evaluated by quantifying CD45^+^ cells, which mark all immune cell populations, including infiltrating T-cells and macrophages^36^. These parameters were analyzed after a 6-day perfusion culture, applied to both Fresh and SF OC tissue samples. αSMA^+^ cells were present in both Fresh and SF-derived cultures with comparable distribution and intensity (range from 2% to 34% for both; Fresh median = 18%; SF median = 8%; *p* = 0.54) (Fig. 3A). These data were confirmed by D2-40 staining, which identified no significant differences in the percentage of the stromal area in the tumor mass between the two groups (range from 40% to 80% for both; Fresh median = 60%; SF median = 62.5%; *p* = 0.92) (Fig. S6A). Notably, the morphology of these stromal cells remained intact in both conditions, displaying the elongated, spindle-like shape characteristic of CAFs. This suggests that the perfusion culture system not only maintains the viability of stromal cell populations but also preserves the typical morphological features after the slow-freezing process. Since D2-40 is also a marker of lymphatic vessels, even though their clinical significance in HGSC is currently uncertain^37^, we were able to occasionally observe them as intact structures after 6-day culture. A representative image of lymphatic vessels in an SF specimen is reported in Fig. S6B.

Regarding vascular integrity, αSMA^+^/CD31^+^ staining revealed the presence of vascular structures in both Fresh and SF tissues (Fig. 3B; Fig. S7).

Quantification of αSMA^+^/CD31^+^ vessels showed no significant difference in their number between Fresh and SF specimens (range from 0 to 34 for both; median = 4 for both; p = 0.88) (Fig. 3B). Overall, vessels were observed in 6/7 Fresh (86%) and 7/8 SF (87.5%) samples, demonstrating that slow freezing seems to permit identifying vascular structures after 6 days of culturing.

Finally, we assessed the ability of the U-CUP perfused system to maintain immune cells within the cultured OC tissues by quantifying CD45^+^ cells. The analysis revealed a variable abundance of immune cells across specimens, with CD45^+^ cells ranging from 2% to 61% in Fresh samples and from 4% to 73% in SF samples (Fig. 3C). Despite such variability, no significant differences in the median percentage (Fresh median = 28%; SF median = 40%) of CD45^+^ cells were observed between the Fresh and SF groups (*p* = 0.31), indicating that the slow-freezing process effectively preserves the immune cells within the TME. These results underscore the robustness of the U-CUP perfusion culture system in maintaining not only the structural integrity of stromal and vascular components but also the immune cell population within the OC TME. Importantly, the ability to preserve immune cell viability and distribution post-freezing further supports the utility of this method for ex vivo studies.

### Response to cisplatin can be efficiently assessed in OC cells under perfusion

In the context of personalized medicine, one of the most key applications of ex vivo cultures is testing the efficacy of existing and novel therapies prior to drug administration, to predict the patient treatment response.

**Fig. 4:**
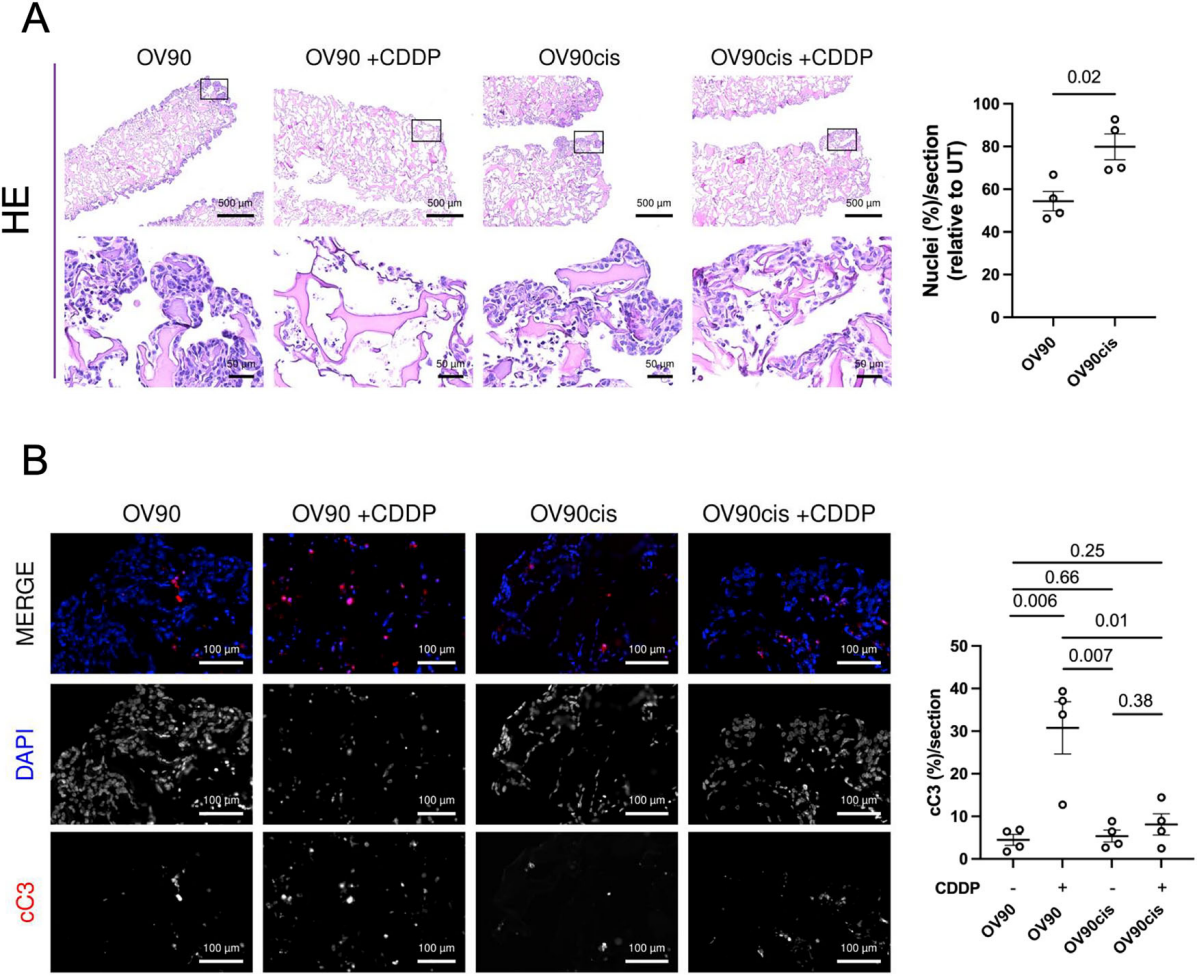
Response to cisplatin can be efficiently assessed in ovarian cancer (OC) cells under perfusion. **A** Representative images of haematoxylin and eosin (HE) staining of the U-CUP perfused sensitive (OV90) and resistant (OV90cis) OC cells treated with cisplatin (CDDP, 18 μM). Black squares indicate the insets of the areas shown at higher magnification. Scatter plot represents the nuclei percentage within the 4 µm section, normalized to the untreated control (UT). **B** Representative IF images of the U-CUP perfused sensitive (OV90) and resistant (OV90cis) cells treated with cisplatin (CDDP, 18 µM). Nuclei (DAPI, blue) and cleaved caspase 3 (cC3, red) are shown in MERGE. The single fluorescence channel is shown separately in black/white images. Scatter plot represents the percentage of cC3^+^ cells per 4 µm section. For all panels, data from two independent experiments (*n* = 2 biological replicates for each experiment) and the mean values ±SEM are shown. QuPath was used for quantification; unpaired t-tests were applied for comparison; and p-values are shown within the graphs.

To demonstrate that the U-CUP is compatible with OC SCT administration and can be used to evaluate the response to this treatment, we utilized a stable cell line-based model of OC. Specifically, we employed the ascites-derived OV90 cells and generated a syngeneic cisplatin (CDDP)-resistant counterpart (OV90cis), enabling us to assess different degrees of platinum cytotoxicity. Both cell cultures were grown in a collagen type I scaffold, providing 3D tissue-like structures for 8 days of U-CUP perfused culture^22,38^. The cisplatin concentration for assessing therapeutic response was determined by measuring the CDDP IC50 of OV90 and OV90cis cells in 2D culture, yielding values of 3 µM and 18 µM, respectively, confirming the CDDP-resistant phenotype of OV90cis cells (Fig. S8B). Since 3D models often exhibit higher IC50 values for various chemotherapeutics, including CDDP^39,40^, we treated both cell lines with a drug concentration corresponding to the IC50 of OV90cis cells, i.e., 18 µM for the last 72 h of culture to observe a clear effect. Post-treatment viability was assessed by comparing nuclei counts per FFPE section, ensuring each section covered the entire scaffold area. CDDP treatment reduced viability by approximately 50% in OV90 cells compared to untreated controls, while OV90cis cells showed a reduction of about 20% (Fig. 4A). Notwithstanding the expected reduced sensitivity to CDDP in 3D culture, the loss of viability was significantly greater in sensitive OV90 cells than in resistant OV90cis cells (p = 0.02), reflecting their corresponding phenotypes (Fig. 4A).

To confirm that the observed viability loss was a specific cytotoxic effect induced by CDDP, we stained sections for the apoptotic marker cleaved caspase 3 (cC3)^41,42^. OV90cis cells showed no significant increase in apoptosis upon CDDP treatment compared to untreated controls (8% versus 5%, respectively; Fig. 4B). In contrast, CDDP induced a significant increase in cC3 positivity in OV90 cells compared to their untreated counterpart (31% versus 5%, respectively; *p* = 0.006) (Fig. 4B). Overall, cC3 positivity was significantly higher in OV90 cells compared to OV90cis after treatment (31% versus 8%, respectively; *p* = 0.01) (Fig. 4B), confirming CDDP specific effect on viability loss. These results demonstrate that U-CUP permits adequate drug delivery and accurately predicts acquired chemoresistance to platinum.

### Perfused OC tissues derived from slow-frozen specimens respond to SCT

**Fig. 5:**
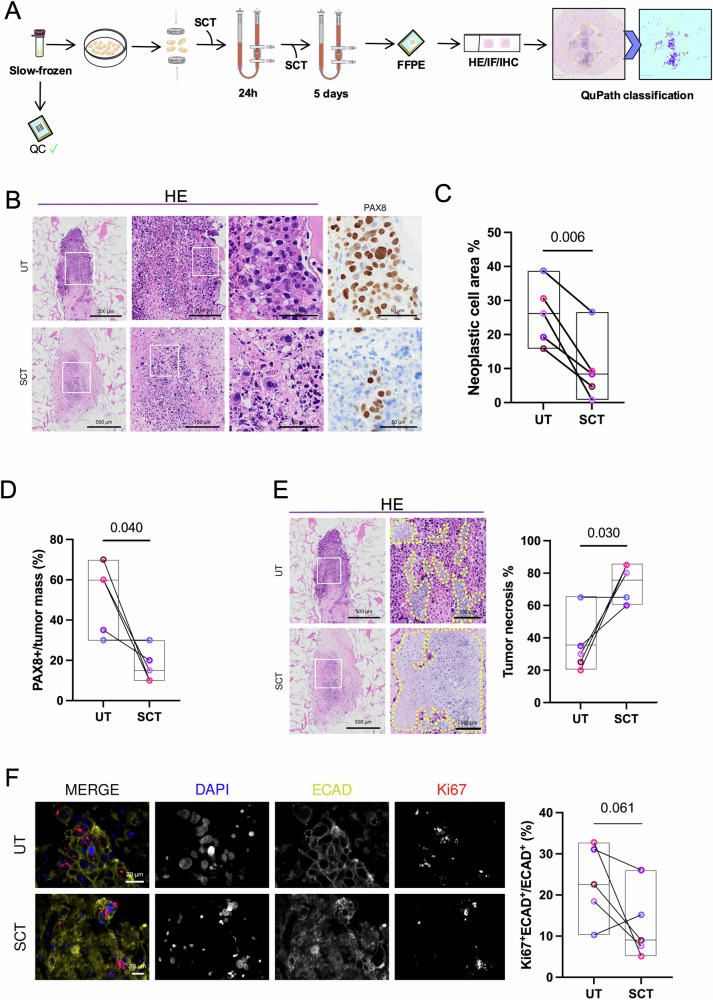
Perfused ovarian cancer (OC) tissues derived from slow-frozen specimens respond to SCT. **A** Schematic representation of the experimental setting. Slow-Frozen chunks derived from HGSC, presenting with ≥10% of neoplastic cell area after 6-day culture, were cultured in U-CUP, in the presence of a combination of carboplatin (70 μM) and paclitaxel (100 nM) mimicking a cycle of standard chemotherapy (SCT), or untreated (UT). After 24 h, all samples were replenished with fresh media without SCT, left in perfused culture for the subsequent 5 days and processed for downstream analyses. **B** Representative images of haematoxylin and eosin (HE) staining and PAX8 immunohistochemistry of UT and SCT-treated OC specimens cultured in U-CUP. **C** Line plot indicates the percentage of neoplastic cell area with respect to the area of the specimen in UT and SCT-treated OC samples. QuPath was used for quantification. **D** Line plot indicates the percentage of PAX8^+^ cells in the tumor mass for each specimen, in untreated (UT) and SCT-treated OC samples. Quantification was performed by a trained pathologist. **E** Representative HE images of necrotic areas (dashed lines) observed in UT and SCT-treated OC samples. Line plot indicates the percentage of necrosis in the tumor mass quantified in paired tissues by a trained pathologist. **F** Representative IF images of UT and SCT-treated HGSC samples. Nuclei (DAPI, blue), epithelial cells (ECAD, dark yellow), and the proliferative marker Ki67 (red) are shown in MERGE. The single fluorescence channels are shown separately in black/white images. Line plot represents the percentage of Ki67^+^ECAD^+^ cells normalized to all ECAD^+^ cells per specimen. QuPath was used for quantification. For all panels, color coding was used to track the cultures deriving from the same patient; floating bar overlay indicates minimum to maximum, and the median value (black line) for each dataset (*n* = 5), paired t-test was applied for comparison and *p*-values are indicated for statistical comparisons.

After validation of the U-CUP bioreactor as a suitable tool for evaluating CDDP response, we were prompted to test SCT efficacy on patient-derived specimens. Indeed, preclinical models for OC encompass not only traditional 2D and 3D models, but also PDO, currently regarded as the most appealing and promising option for in vitro testing^43^. However, a limitation of OC PDO is their inability to fully recapitulate the entire human TME, posing challenges when translating related findings to clinical contexts^11–13^. Given the pressing demand for more reliable models for therapy testing, we assessed the effectiveness of employing ex vivo cultures perfused in U-CUP bioreactor as an innovative preclinical model for OC outcomes. The first line of treatment for OC patients typically involves either neoadjuvant or adjuvant chemotherapy, using carboplatin and paclitaxel. To understand whether U-CUP allows measuring the response to SCT treatment in patient-derived tissue, we cultured SF OC specimens derived from 5 chemo-naïve patients (Table S1). SF specimens with initial neoplastic cell area >30% were chosen, to ensure the high success rate of the experiment. Each culture was treated with either vehicle or the combination of carboplatin and paclitaxel, using drug concentrations which are deemed clinically significant in patients, to mimic one cycle of patient SCT administration^44,45^. After 24 h of treatment drugs were washed out and cultures maintained for 5 days before processing for downstream analyses (Fig. 5A). An overall reduction in neoplastic cell area and PAX8^+^ cells was observed after SCT administration compared to the corresponding untreated (UT) specimens (UT median 26%, SCT median 8%, p = 0.006; and UT median 60%, SCT median 15%, p = 0.04, respectively) (Fig. 5B–D). Moreover, to verify that such a decrease resulted from a specific cytotoxic effect induced by SCT administration, we next measured necrotic cell death and Ki67 as readout of SCT effect. Tumor necrosis was significantly increased upon SCT treatment (UT median 30%, SCT median 80%, *p* = 0.03) (Fig. 5E), suggesting that the perfusion of SCT in U-CUP effectively induced cytotoxicity, impacting cell viability in the analyzed ex vivo samples. Finally, to determine whether SCT administration on perfused ex vivo specimens could affect tumor cell proliferation, we assessed the mitotic index in cancer cells, and a trend of reduced proliferation was observed in the SCT-treated OC tissues (UT median 23%, SCT median 9%, *p* = 0.061). (Fig. 5F).

Taken together, these data demonstrate U-CUP system is a valuable tool for assessing SCT-induced tumor regression in patient-derived tumor tissue. Of note, variable degrees of response were observed in different patients. In particular, the decrease in neoplastic cell area ranged from 3% to 69%, indicating a potential difference in sensitivity to treatment among samples (Fig. S9). Moreover, IHC staining for PAX8 revealed a decrease in the percentage of viable tumor cells after treatment, in all but one specimen. Interestingly, the same sample retained a higher number of PAX8^+^ tumor cells, showed a reduced extent of tumor necrosis, and exhibited a comparable mitotic index to the corresponding UT (Fig. 5D–F), indicating a potential degree of resistance following treatment in this sample. These findings suggest that the perfusion-based culture method for ex vivo culturing shows potential for testing pharmacological treatments, with varying success rates contingent upon tumor biology.

## Discussion

Current therapies for OC face significant challenges, primarily due to the development of resistance and the resulting high recurrence rates, which are the leading causes of mortality^5,6^. Additionally, one major challenge is the lack of reliable predictors for determining the success of treatment regimens. Ex vivo assays, such as the one presented in our study, could address this gap by providing a platform to evaluate and or predict the effectiveness of therapies prior to their administration, particularly for neoplasms such as HGSC, for which the time interval between surgery and the beginning of treatment is about 29 days (interquartile range 24;37)^46^.

The complexity of the TME plays a key role in therapy resistance^7^. Our study demonstrates that the U-CUP perfusion bioreactor effectively preserves both cancer cell viability and the integrity of key TME components, whether Fresh or Slow-Frozen. This preservation is crucial for modeling complex in vivo conditions and holds significant potential for advancing translational research and personalized medicine. Indeed, traditional culture systems often fail to replicate the complexity of the TME, leading to the loss of essential stromal and immune components^10,34^. The superior preservation of neoplastic cells and tissue architecture in perfusion-based models compared to static 3D cultures further underscores the advantages of this approach, as shown in previous work on other tumor types^19,21^.

The extracellular matrix and the dynamic in vivo microenvironment are key factors in tissue engineering and cancer research. Lutolf and Hubbell^47^ demonstrated that bioengineered scaffolds can emulate the extracellular matrix in 3D cultures, influencing cell behavior and function. Similarly, Mendoza-Martinez et al.^48^ stress the need for advanced 3D models that replicate not only cancer cells but also the diverse cellular and extracellular matrix components that influence tumor progression and drug resistance. By better preserving the original TME the U-CUP bioreactor provides an essential model for studying OC biology and treatment responses^48,49^.

Dynamic mechanical forces, such as shear stress, are also critical for maintaining tissue viability and function. Huh et al.^50^ demonstrated that perfusion-based systems preserve tissue functionality by replicating these forces, which mirrors our findings on the ability of the U-CUP system to maintain cancer cell viability and proliferation. As highlighted by Lopez et al.^51^, incorporating dynamic mechanical factors is essential for accurately modeling the in vivo behavior of OC cells, potentially enabling better prediction of therapeutic responses. These studies collectively support our approach, underscoring the potential of dynamic culture systems for drug testing and personalized medicine.

The maintenance of cell viability and proliferative capacity in slow-frozen tissues addresses a significant limitation in translational research, where access to fresh tissue is often restricted. While freezing can compromise cellular integrity, our study indicates that slow-freezing followed by perfusion-based culture preserves neoplastic cell viability and growth potential, as demonstrated by key biomarkers such as PAX8 and Ki67^29^. This is a significant advancement for the creation of tissue biobanks and future therapeutic studies. Interestingly, a few cases exhibited increased neoplastic tissue area after 6 days in culture compared to their corresponding d0 samples. While this may reflect randomization-related heterogeneity, it could also suggest that certain highly proliferative tumor samples maintain their growth potential in ex vivo culture.

Further analysis through αSMA and CD31 staining confirmed that the U-CUP bioreactor not only maintains cellular viability but also preserves key stromal and endothelial components. This is particularly important for studying tumor-stroma interactions, which are known to influence therapeutic response and tumor progression in OC^10,34^. The ability of the U-CUP system to maintain vascular integrity and immune cell viability, even in slow-frozen tissues, expands its utility for investigating angiogenesis and immune cell infiltration. Given that poor responses to immunotherapy in OC have been largely attributed to tumor heterogeneity^52^, the need for more representative and reliable models is cogent in the attempt to identify novel biomarkers and testing new therapies to evaluate their preclinical efficacy. Preserved TME components allow for more accurate drug response studies, which is pivotal in assessing patient-specific reactions to chemotherapy. Our study reveals variability in chemotherapy responses among different patient samples, as observed in the differential reduction of neoplastic cell areas and the proliferative index post-treatment. This variability underscores the heterogeneity of OC and highlights the importance of patient-derived models for personalized therapy testing. As noted by Lopez et al.^51^ and others^14,21^, the heterogeneity of HGSC and the influence of the microenvironment on drug responses necessitate models that closely mimic in vivo conditions. The patient-derived OC models developed in this study offer the potential to optimize individual therapies by improving our ability to predict treatment responses based on the specific characteristics of each patient’s tumor.

Furthermore, the limited success of immune checkpoint inhibitors, such as avelumab, in OC treatment highlights the need for more accurate ex vivo models. Clinical trials have shown no significant improvement in progression-free survival or overall survival with avelumab in advanced-stage or platinum-resistant OC patients^53–55^. Our perfusion-based culture system, as indicated by CD45 staining, suggests that some immune cell populations are preserved, offering a platform for further investigation into immune mechanisms within the TME and their role in therapeutic resistance.

However, the system is not without limitations. The observed necrosis in both fresh and frozen tissues after six days suggests that further optimization of perfusion parameters, such as flow rates or nutrient composition, may be necessary to reduce cell death in tissue cores and extend tissue viability. While slow-freezing preserved most TME elements, subtle morphological changes in pericytes associated with endothelial cells suggest that fresh tissues may still be preferable for angiogenesis-focused studies. Optimizing cryopreservation protocols could improve the preservation of TME integrity, particularly in long-term studies.

Relying on patient-derived material presents challenges for drug screening, primarily due to limited tissue availability. Strategies such as the amplification of patient-derived cells, including PDOs and PDXs, could increase material for testing, though they may introduce phenotypic changes over time due to in vitro culture conditions or host interactions^56,57^. Integrating microfluidics into culture systems could reduce the required sample size and enhance assay efficiency^58,59^. Mendoza-Martinez et al.^48^ similarly advocate for the development of advanced 3D systems, such as microfluidic and hydrogel-based platforms, to improve the fidelity of in vitro models to in vivo tumor conditions.

This study demonstrates that the U-CUP bioreactor effectively supports the ex vivo culture of OC tissues, preserving both neoplastic cells and essential components of the TME from SF specimens. Our results provide clear evidence that perfusion-based systems outperform static cultures in maintaining tissue architecture and cellular viability, making them highly valuable for studying tumor biology and therapeutic responses. The preservation of TME elements, including stromal and immune cells, further solidifies this model utility in translational research, particularly for personalized medicine and therapy evaluation.

By leveraging frozen tissue samples from biobanks, this approach overcomes significant barriers related to tissue availability, offering a practical solution for retrospective drug screening and preclinical testing. This allows the exploitation of these underutilized resources, enabling perspective and retrospective re-evaluations of samples under different therapeutic regimens. Future studies should focus on optimizing perfusion parameters to reduce necrosis, which is essential for maintaining tissue viability and improving the model accuracy in predicting chemotherapy responses. Expanding the sample size to include patients with neoadjuvant treatments and relapses will further strengthen the predictive power of OC U-CUP cultures.

By aligning with established principles of tissue engineering and dynamic culture systems^60,61^, the U-CUP bioreactor holds substantial promise for advancing our understanding of tumor biology and developing more personalized therapeutic strategies, offering a valuable tool for both academic research and clinical applications.

## Methods

### OC tissue collection and preparation for ex vivo culture

Tumor samples were collected from OC patients who underwent upfront surgery (i.e., without prior neoadjuvant chemotherapy or other treatments) as part of standard clinical care. Only patients diagnosed with tubo-ovarian high-grade serous carcinoma (HGSC), confirmed by histological examination, were included, with the exception of one low-grade serous carcinoma (LGSC, OC-3) sample. This study was approved by the following ethics committees: the Ethikkommission Nordwest- und Zentralschweiz (EKNZ; Swiss ethics approval system BASEC IDs: 2017–01900, 2023-00988) and the Comitato Etico Area Vasta Emilia-Romagna Centro (CE-AVEC; approval ID: MIPEO EM109-2020_107/2011/U/Tess/AOUBo). Written informed consent was obtained from all patients prior to surgery, and the study was conducted in accordance with the Declaration of Helsinki. Both primary and metastatic samples (omental or peritoneal) were used for treatment experiments in static in vitro culture, perfusion bioreactor experiments, or cryo-conservation immediately after surgery. In total, 19 tumor samples derived from 17 OC patients were included in the study (Table S1).

The OC specimens were preserved in MACS Tissue Storage Solution (TSS) at 4 °C, supplied by Miltenyi Biotec, and were used within 24 h. For preparation, the specimens were divided into small 2 × 2 × 2 mm^3^ pieces using a scalpel. This size was chosen to optimize the perfusion flow within the bioreactor chamber and to reduce tissue damage during the cutting process. The tissue chunks were then randomized by gentle shaking in TSS and either processed as “Fresh” material for immediate experimental use or slowly frozen for storage in a freezing medium using a standard, controlled-rate cryopreservation protocol consisting of pure fetal bovine serum (FBS) with 10% dimethyl sulfoxide (DMSO). The frozen samples were first stored at -80 °C for at least 24 h in a freezing container before being transferred to long term storage in liquid nitrogen.

### Ex vivo tissue culture and treatments

Three to five tumor pieces were sandwiched (Patent n. WO2015181185A1) between two 10 mm in diameter discs of type I collagen (Ultrafoam collagen haemostat from Davol, Inc.) and assembled in the U-CUP perfusion chamber (Cellec Biotek AG) according to the manufacturer’s instructions. The bioreactors were then filled with 8 mL of Advanced DMEM/F12 medium supplemented as described in Table S5 (adapted form^62^). A syringe pump system (Programmable Harvard Apparatus PHD ULTRA 2000, Cellec Biotek AG) was connected to the bioreactors to maintain a bidirectional flow rate of 0.47 mL/min. As a control, tumor tissue pieces were also cultured in a static petri-dish-like culture with the same media, as previously described^19–21^. All cultures were maintained in a humidified atmosphere with 5% CO2 at 37 °C.

To mimic the administration of one SCT cycle, 3D ex vivo cultures were treated with 70 µM and 100 nM of carboplatin and paclitaxel, respectively, for 24 h. The treatment was then removed, replenishment with fresh medium was performed, and the culturing continued for an additional 6 days before downstream analysis. Only slow-frozen chunks with ≥10% of neoplastic cell area after 6 days of culture were cultured in the U-CUP to mimic SCT.

### Cell lines

The human OC OV90 cell line was purchased from the ATCC (American Type Culture Collection), and cultured at 37 °C in an incubator with 5% CO_2,_ using RPMI 1640 Medium (Life technologies #31870-025) supplemented with 10% FBS (Life Technologies #10270), 1% Penicillin/Streptomycin (Life Technologies #15070-063) and 2 mM L-Glutamine (Lonza BE #17-605E). The cells were authenticated by AmpFlSTR Identifiler PCR Amplification kit (Applied Biosystems #4322288) (Fig. S8A), and regularly tested for mycoplasma contamination upon thawing and prior to the experiments. The cisplatin (CDDP)-resistant OV90 cell line (OV90cis) was generated by three cycles of exposure of the OV90 parental sensitive cell line (OV90) to cisplatin at a concentration equal to its IC50 at 72 h (∼3 µM), followed by 72 h in cisplatin-free medium. At the end of the three cycles the remaining viable cells were allowed to grow until visible colonies were formed. Three single colonies were isolated as independent cell lines and further treated for 6 months, alternating between 72 h treatments with CDDP and 72 h CDDP-free medium. With every two cycles, the CDDP concentration was gradually increased by 1 µM per cycle, ultimately reaching ∼20 µM final concentration. The OV90 cell lines were treated periodically with CDDP at its IC50 every four passages to avoid a resistant phenotype reversion.

### Determination of IC50 in 2D

The half-maximal inhibitory concentrations (IC50) of CDDP for OV90 and OV90cis in 2D were assessed after 72 h of treatment with CDDP (concentrations ranging from 1 to 64 µM), using the MTT viability assay (Fig. S8B). Briefly, OV90 and OV90cis were seeded in a 96-well plate (3000 cells/well). After 72 h of growth 20 µL of 5 mg/mL Thiazolyl Blue Tetrazolium Bromide (MTT) solution (Sigma-Aldrich, # M2128) was added to each well, and the plates were incubated at 37 °C for 3 h. After the removal of the medium, the precipitate was solubilized in 200 µL of DMSO, and the absorbance was measured at 540 nm using a BioTek Synergy H1M plate reader.

### 3D cell culture establishment under perfusion and treatments

To obtain 3D tissue-like structures of OV90/OV90cis cells, they were seeded 2.6 × 10^6^ on an 8 mm in diameter x 4 mm height collagen type I scaffold (Ultrafoam collagen haemostat from Davol, Inc.) and assembled in the perfusion chamber according to the manufacturer’s instructions. The bioreactors were then filled with 10 mL of cell culture medium as described above. The syringe pump system (Programmable Harvard Apparatus PHD ULTRA 2000) was connected to the bioreactors to maintain a bidirectional flow rate of 3.00 mL/min for 48 h. Then, the flow rate was changed to 0.25 mL/min for 72 h. All cultures were maintained in a humidified atmosphere with 5% CO2 at 37 °C. Cell lines were then treated with 18 µM of CDDP at a flow rate of 0.25 mL/min for an additional 72 h.

### Histology analyses

The tissue chunks-scaffold constructs, each containing 3 to 5 randomized tissue chunks, were formalin-fixed and paraffin-embedded following standard protocols. During embedding, the perfused samples were positioned to allow longitudinal sectioning of the collagen-tissue-collagen sandwich. To ensure representativeness and minimize sample bias, 1–3 different sections per chunk were analyzed at varying depths, with 40 µm spacing between sections. Sections of 4 μm thickness were stained with HE following the standardized protocol (Epredia™ Gemini™ AS Automated Slide Stainer). For PAX8 immunohistochemistry Leica BOND RX Research (Leica Biosystems) immunostainer was used and heat-mediated antigen retrieval (HMAR) was performed with ER2 buffer (EDTA-based buffer with basic pH) at 100 °C for 20 min and subsequent antibody incubation (PAX8 clone 1F8-3A8, Thermo Fisher Scientific #MA1-117), diluted in Primary Antibody Diluent (Leica Biosystems, #AR9352), at room temperature for 30 min. All the automatized steps and visualization were performed with the Bond Polymer Refine Detection kit (Leica Biosystems, #DS9800). For CD45, immunohistochemistry BenchMark ULTRA (Ventana, USA) immunostainer was used and HMAR was performed with Ultra Cell Conditioning solution 1 (CC1) (Tris-based buffer with basic pH, #05424569001) at 95 °C for 24 min and subsequent antibody incubation (CD45 clone 2B11&PD7/26, Cell Marque, USA, #760-4279) at 36 °C for 12 min. The visualization was performed by OptiView DAB Detection Kit (Ventana, USA, #06396500001). Podoplanin (D2-40) expression was evaluated using the mouse monoclonal anti-Podoplanin (RTU, Clone D2-40, Cell Marque, USA) with the following protocol: Antigen Retrieval: Ultra CC1 for 32 min at 95 °C; primary antibody incubation: 32 min at 36 °C; visualization with OptiView DAB Detection Kit (Ventana-Roche). IF staining of Ki67, ECAD, cC3, alpha SMA, and CD31 was performed using antibodies and conditions listed in Table S3; blocking was performed with goat serum (Abcam #156046) for 10 min at RT, and the slides were mounted with Vectashield Antifade Mounting Medium with DAPI (Vector Laboratories, #H-1200).

### Image analysis and quantification

The quantitative analysis of the neoplastic cell area percentage in the specimen was performed using the image analysis software QuPath (release 0.4.3)^63^. In detail, images of HE stained tissue sections or 3D cell cultures were acquired with a slide scanning device (Olympus VS200 or Hamamatsu Nanozoomer S60, objective 40x, NA 0.75), and QuPath was used for analysis. For each analysis, 1–3 longitudinal sections of the sandwich were analyzed, and the mean value was considered. The entire tissue area was selected and annotated as the region of interest (ROI) for quantification, except for the Ki67 staining. For Ki67 quantification, the ROI consisted of ‘hot spot’ areas with particularly high Ki67 expression, as these regions reflect the most proliferative areas of the tumor, which are considered relevant for disease outcome^64,65^. To generate the QuPath annotation for recognition of the neoplastic cell area, only the regions prevalently occupied by cancer cells were included, excluding tumor stroma and the adjacent normal tissue of the specimen (Fig. S1A). QuPath annotation was reviewed and confirmed by a trained pathologist. Quantification of cell structures and Ki67/ECAD, cC3, αSMA, and CD31 IF, as well as the immunohistochemical staining for CD45, was also performed using QuPath. Specifically, the StarDist2D algorithm for object detection^66^ or the Watershed cell detection method was used for HE and IF staining, while the Positive Cell Detection algorithm was employed for immunohistochemistry. For IF analyses, only DAPI-positive cells were annotated to be quantified. All scripts and classifiers used for each type of analysis are listed in Table S6. Positive staining was determined by specifying a threshold between positive and negative signals for each analysis separately. An example of a QuPath mask used for the evaluation of each marker is shown in Fig. S1B-C. PAX8 positivity and the extent of tumor coagulative necrosis, as well as the percentage of chemotherapy-induced tumor necrosis, were quantified by a trained pathologist in 1–2 entire longitudinal sections of the specimen. In detail, tumor mass was first defined by including cancer cells and associated tumor stroma, while excluding the scaffold and any adjacent normal area present in the specimen, such as adipose tissue. Next, the percentage of vital PAX8^+^ cells and the percentage of necrotic area in the tumor mass were determined. To generate the QuPath annotation for measuring the neoplastic nuclei area, sections stained for PAX8 were analyzed. Five regions of interest (ROIs) were selected and annotated using the Cellpose Detection model for quantification in 1–3 different sections per chunk (Table S6). The nuclei diameter was then calculated based on the obtained area values (Table S7).

The percentage of podoplanin-stained stromal area in the tumor mass was evaluated by a trained pathologist in one field of view at low magnification (4x). The quantitative analysis of the 3D tissue-like structures obtained by culturing OV90/OV90cis cells was performed by counting the nuclei. The entire area of the section was selected and annotated as the region of interest (ROI) for quantification. An example of a QuPath mask used for counting the nuclei of OV90/OV90cis cells is shown in Fig. S1D.

### DNA extraction and multi-gene NGS sequencing for the identification of somatic mutations

DNA was extracted from uncultured tissue and corresponding perfused cultures derived from Fresh or SF specimens using GenElute™ Mammalian Genomic DNA Miniprep Kits following the manufacturer’s instructions and quantified using the NanoDrop2000. A previously published NGS lab-developed multi-gene panel was used for sequencing^67^, which includes the genomic regions described in Table S4 (human reference sequence hg19/GRCh37, total of 330 amplicons, 21.77 kb). In total, 10 specimens from 5 patients were analyzed (Table S1). Briefly, about 30 ng of input DNA was used for NGS libraries preparation with the AmpliSeq Plus Library Kit 2.0 (Thermo Fisher Scientific). Templates were then sequenced using an Ion 530 chip, and the results were analyzed with the Ion Reporter tool (version 5.16, Thermo Fisher Scientific) and IGV software (Integrative Genome Viewer version 2.12.2 - https://software.broadinstitute.org/software/igv/). According to the previously reported validation^30^, only mutations present in at least 5% of the total number of reads analyzed and observed in both strands were considered for mutational calls. The ACMG classification of detected variants was evaluated using the Varsome database (https://varsome.com/).

### Graph preparation and statistical analysis

GraphPad Prism version 9.5.1 (Boston, Massachusetts, USA, www.graphpad.com) was used for graphical data representation and statistical analyses. The Shapiro-Wilk test was employed to assess the normality of the data distribution, and the F-test was used to evaluate the homogeneity of variances. Unpaired and paired t-tests were used to compare independent and dependent groups, respectively. If the data followed a normal distribution and showed equal variances, a t-test was utilized. Otherwise, the Mann-Whitney and Wilcoxon signed-rank tests were used instead, for independent and paired groups, respectively. Unless stated otherwise, a two-tailed test was applied.

## Supplementary information

Supplementary figures and legends

Supplementary tables

## Data Availability

The datasets generated and/or analyzed during the current study are available in the AMSActa Institutional Research Repository (AlmaDL, University of Bologna Digital Library) at 10.6092/unibo/amsacta/8274.
