# Supplementary material for: Perfusion-based ex vivo culture of frozen ovarian cancer tissues with preserved tumor microenvironment
Source: NPJ Precis Oncol. 2025 May 23;9:152. doi: 10.1038/s41698-025-00941-6 (PMC12102267; doi:10.1038/s41698-025-00941-6)
Supplement: Supplementary file 1 — Supplementary figures and legends [file 41698_2025_941_MOESM1_ESM.docx]

# **Supplementary figures and legends**


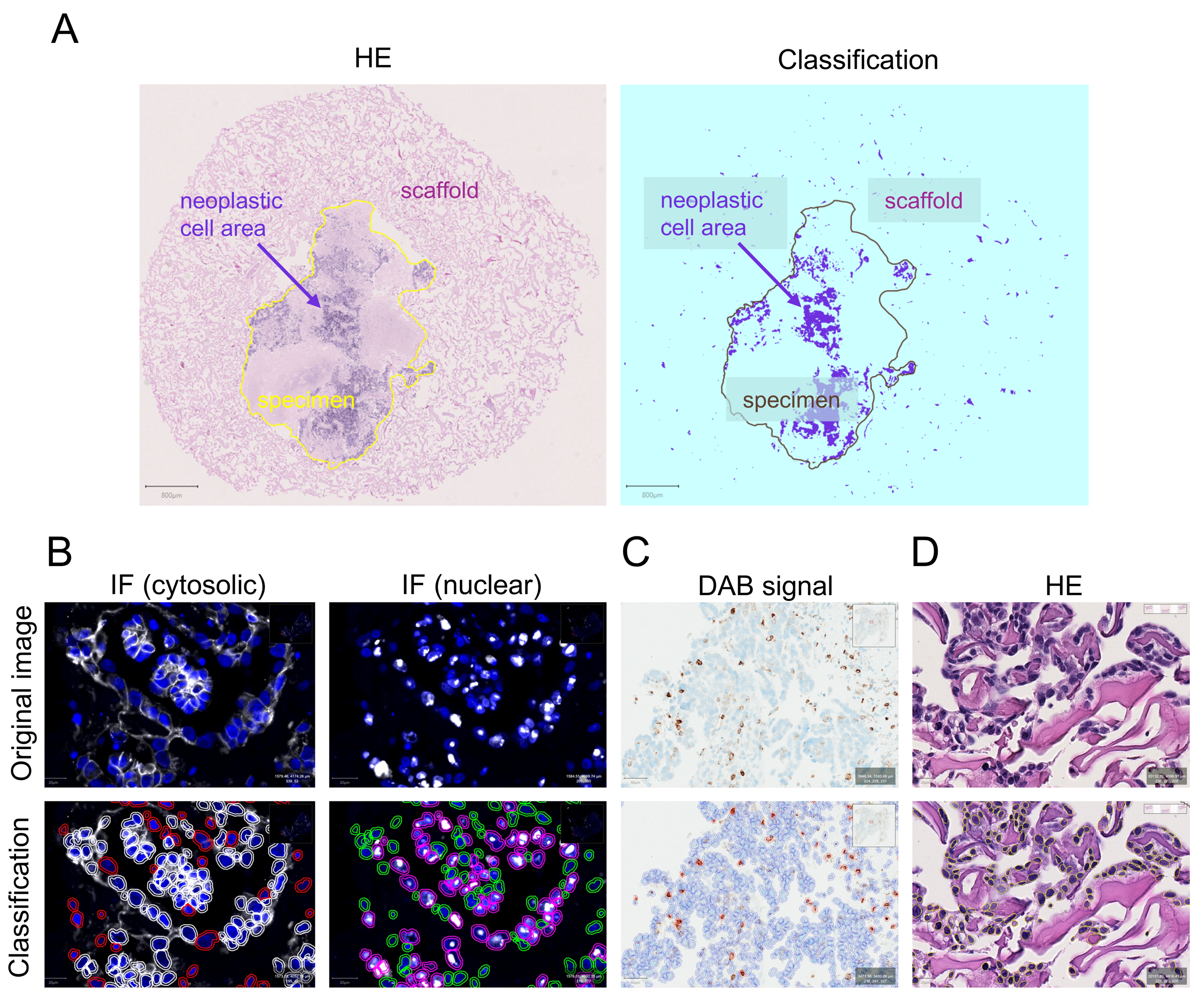


**Figure S1.** **QuPath annotations for image analyses**. **A**. Representative image of haematoxylin and eosin (HE) staining of Pd6 culture and corresponding QuPath pixel classifier (Classification) applied for the recognition of neoplastic cell area (purple) inside the specimen. **B**. Representative immunofluorescence (IF) images and corresponding QuPath object detection and classification used to count cells with cytosolic/nuclear positive (white/magenta circles) and negative (red/green circles) signals. **C**. A representative immunohistochemistry image of anti-CD45 staining and corresponding QuPath annotation used to count 3,3'-diaminobenzidine (DAB) positive cells (red). **D**. Representative image of HE staining of perfused 3D OV90 cell cultures and corresponding QuPath detection and classification used to count nuclei (yellow).

**
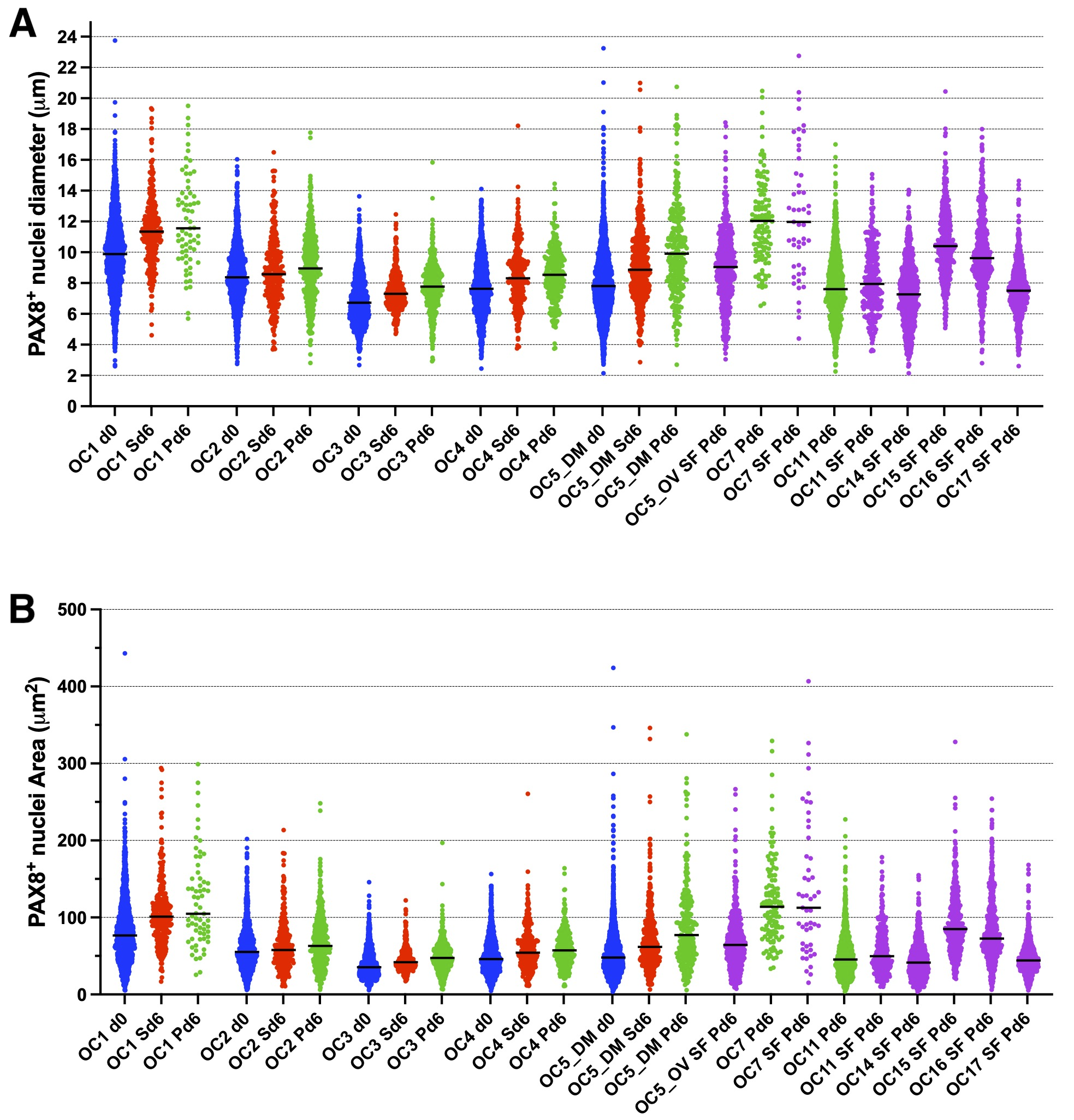
**

**Figure S2. Analysis of nuclear size in PAX8^+^ tumor cells.** Scatter dot plots showing the distribution of individual nuclear measurements, diameter (**A**) and area (**B**), in uncultured Fresh HGSC and the LGSC OC-3 tissues (d0, blue dots, n=5) and their paired counterparts cultured under static conditions (Sd6, red dots, n=5) or perfused (Pd6, green dots, n=5). Additional unpaired samples include Fresh (Pd6, green dots, n=2) and Slow frozen (SF, violet dots, n=7) HGSC specimens. Median values are indicated by black lines. QuPath was used for quantification.


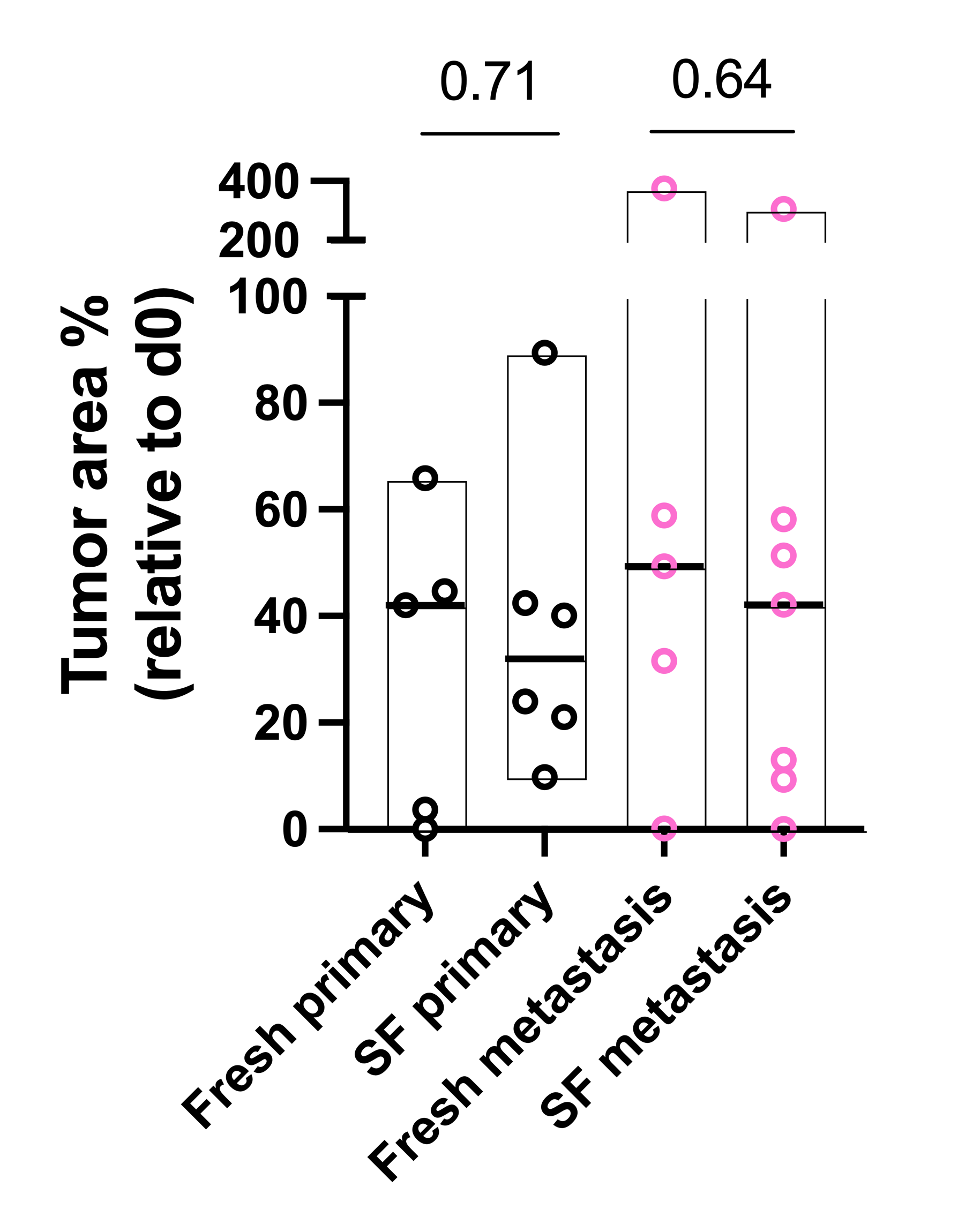


**Figure S3. Neoplastic cell area in Fresh and Slow frozen perfused OC specimens.** Cultures derived from Fresh HGSC and the LGSC OC-3 (n=5 primary, n=5 metastasis) and Slow frozen (SF, n=6 primary, n=7 metastasis) HGSC specimens. Black and pink circles indicate primary tumors and metastases, respectively. Floating bar overlay indicates minimum to maximum, and the median value (black line) for each dataset. QuPath was used for quantification. Unpaired t-test was applied for group comparison; p-value is shown within the graph.


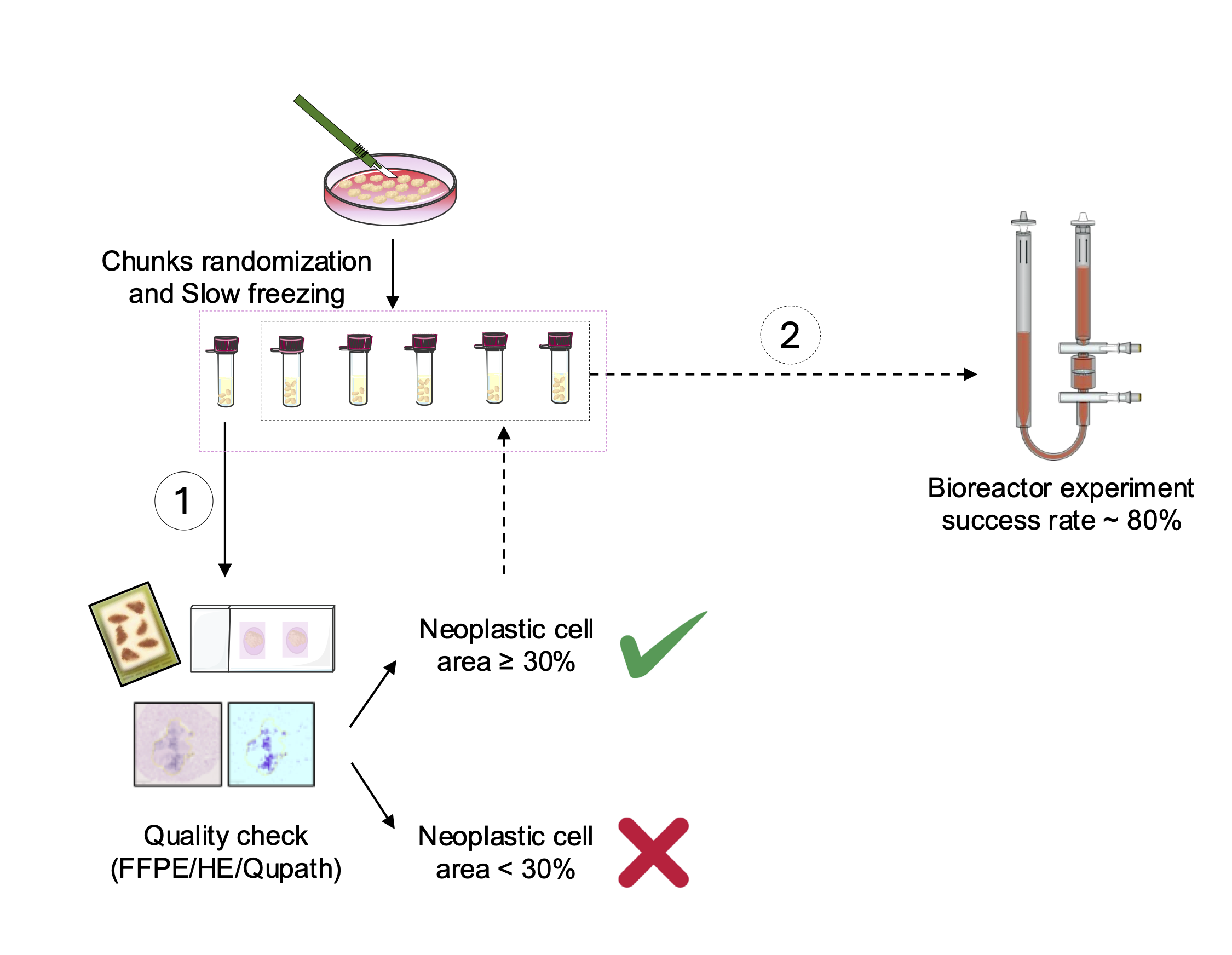


**Figure S4**. **Slow-Frozen sample quality check workflow**. Specimen is cut into chunks (2x2x2 mm^3^), which are randomized and then slow-frozen by placing 4-6 chunks in the quality check vial and 8-10 chunks/vial for experimental procedures. (1) Before setting up the experiment, the chunks from the quality check vial are thawed and formalin-fixed/paraffin-embedded (FFPE). Haematoxylin and eosin (HE) staining is performed to allow the QuPath mediated quantification of neoplastic cell area. (2) Only those samples in which ≥ 30% of the neoplastic area is observed are used for the bioreactor experiments. Following this quality check, there is 80% probability that the specimen cultured in perfusion for 6 days maintains sufficient viable cells (≥ 10%) required for robust downstream analyses.


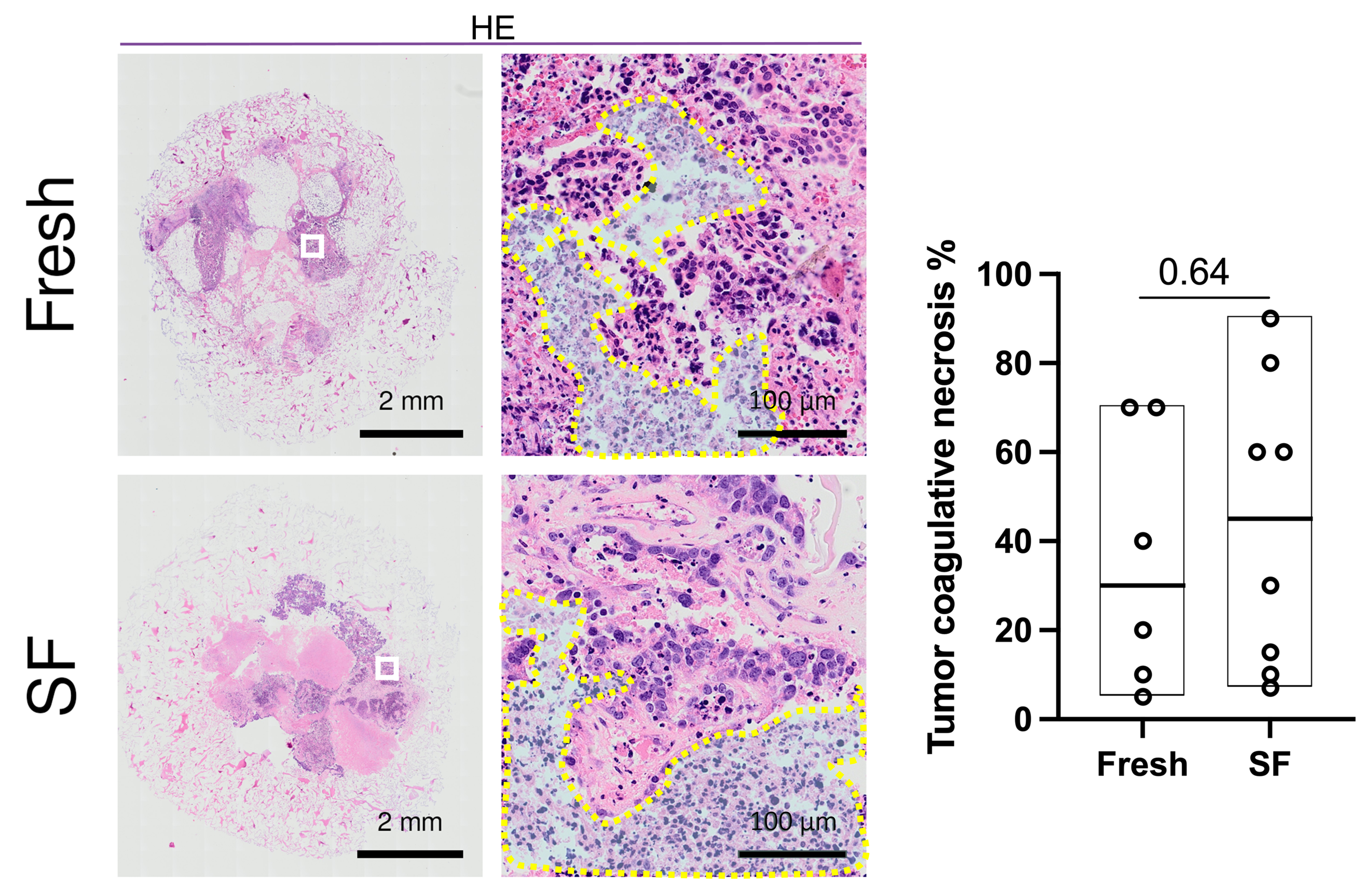


**Figure S5.** **Necrosis assessment in Fresh and Slow-Frozen cultures**. Necrosis in cultures derived from Fresh (n=6) and Slow-Frozen (SF, n=8) HGSC specimens. Representative images are shown of haematoxylin and eosin (HE) staining. White squares indicate the insets of the areas shown at higher magnification and dashed lines indicate necrosis. Floating bar overlay indicates minimum to maximum, and the median value (black line) for each dataset. Quantification was performed by a trained pathologist. Unpaired t-test was applied for comparison and p-value is shown within the graph.


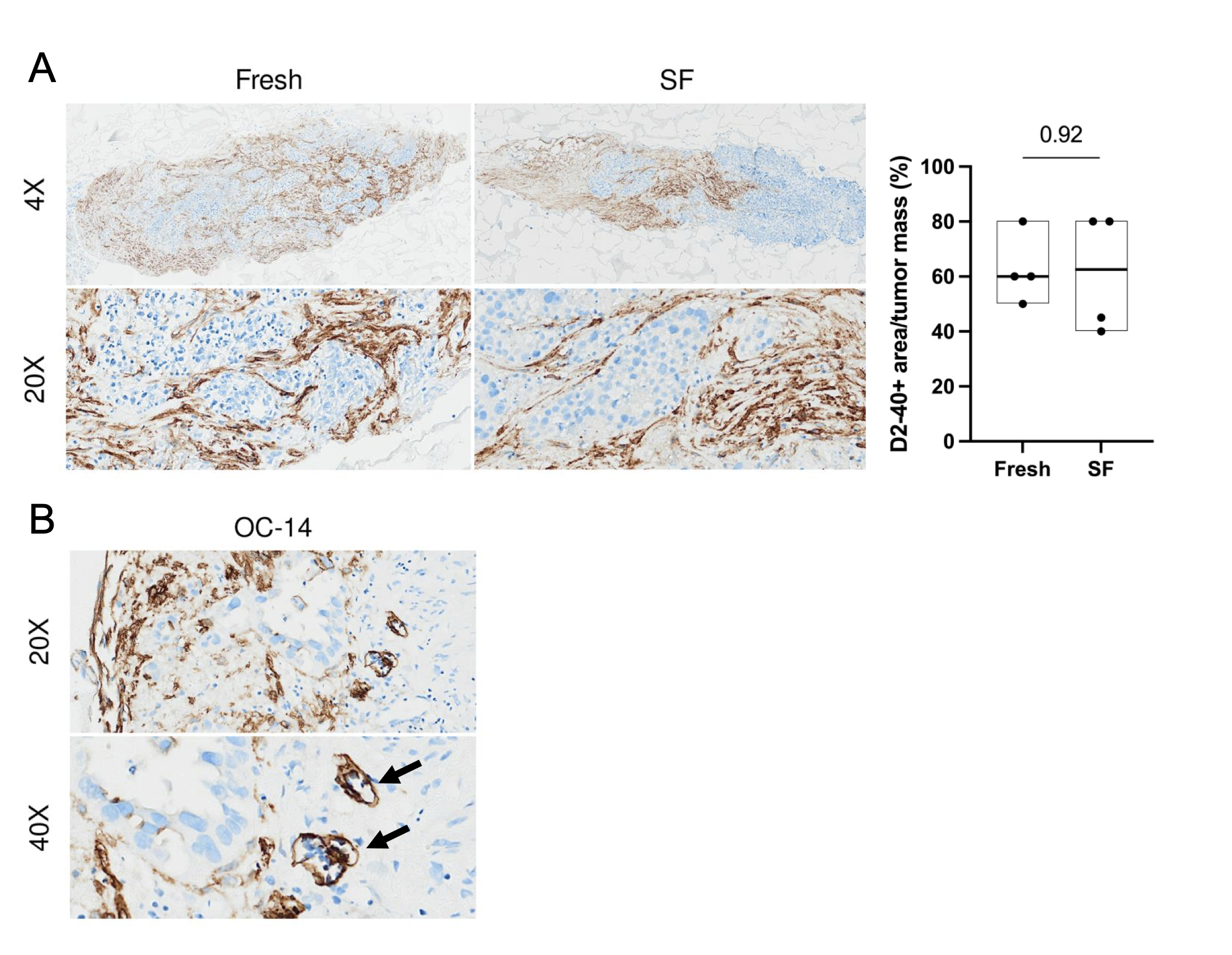


**Figure S6. D2-40 immunohistochemistry in perfused cultures derived from Fresh or Slow-Frozen tissues. A.** Representative images of Fresh and SF HGSC samples stained for D2-40 marker. Floating bar graphs indicate D2-40+ area relative to tumor mass (specimen). Minimum, maximum, and median values (black line) for each dataset are shown (Fresh, n=4; SF, n=4). Quantification was performed by a trained pathologist, unpaired t-test was applied for comparison and p-value is shown within the graph. **B.** Representative image of D2-40 staining in OC-14 SF sample presenting with lymphatic vessels (arrows).


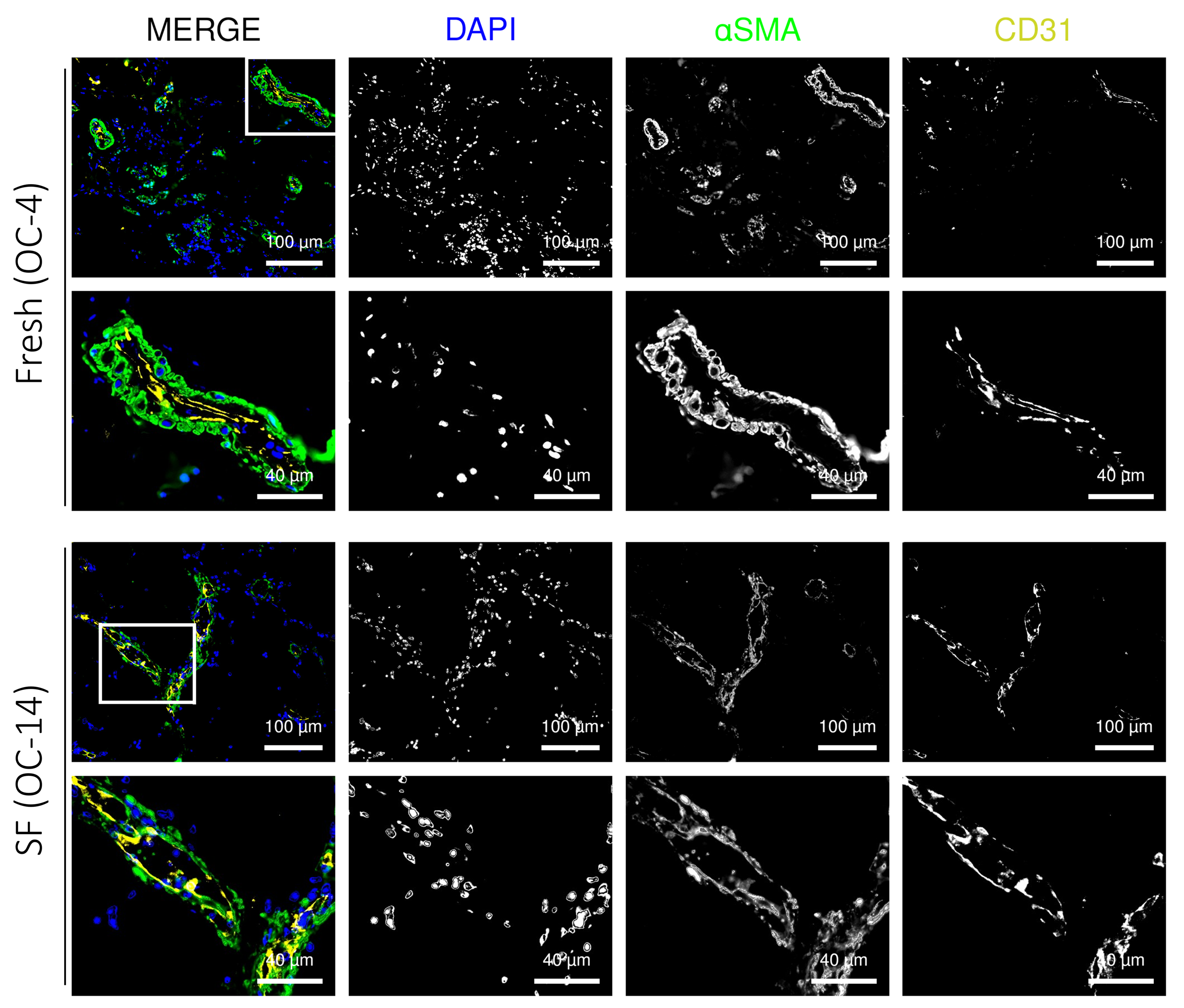


**Figure S7.** **Immunofluorescence characterization of vessels in Fresh and Slow-Frozen cultures**. Representative immunofluorescence images of perfused cultures derived from Fresh (OC-4) and SF (OC-14) tissues. Nuclei (DAPI), the pericyte marker ⍺SMA (green) and the endothelial marker CD31 (yellow) are shown in MERGE and the corresponding black and white channels. White squares indicate the insets of the areas shown at higher magnification.


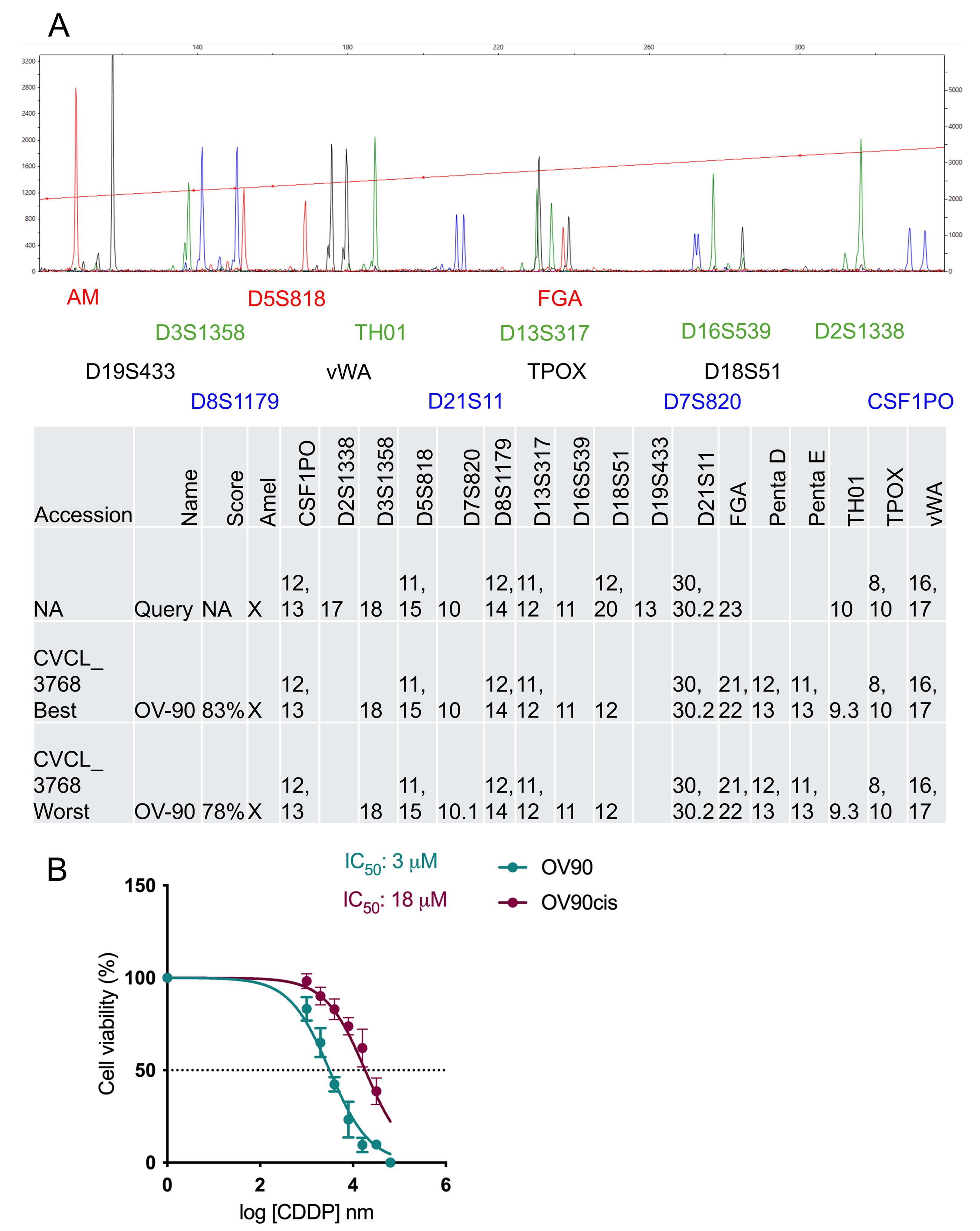


**Figure S8.** **Generation and characterization of the OV90 sensitive and resistant cell lines**. **A**. Peak Scanner chromatograms of the AmpFlSTR Identifiler analysis and Cellosaurus output of microsatellite comparison performed to authenticate OV90 cell line (Query). **B**. Dose-response curves for 72h cisplatin (CDDP) treatment of sensitive (OV90) and resistant (OV90cis) OC cells grown in 2D. Half maximal vitality inhibitory CDDP concentrations are indicated (IC50). Cell viability was determined by MTT assay. Data (mean ± SEM, n=3) are expressed as a percentage of untreated cells viability.


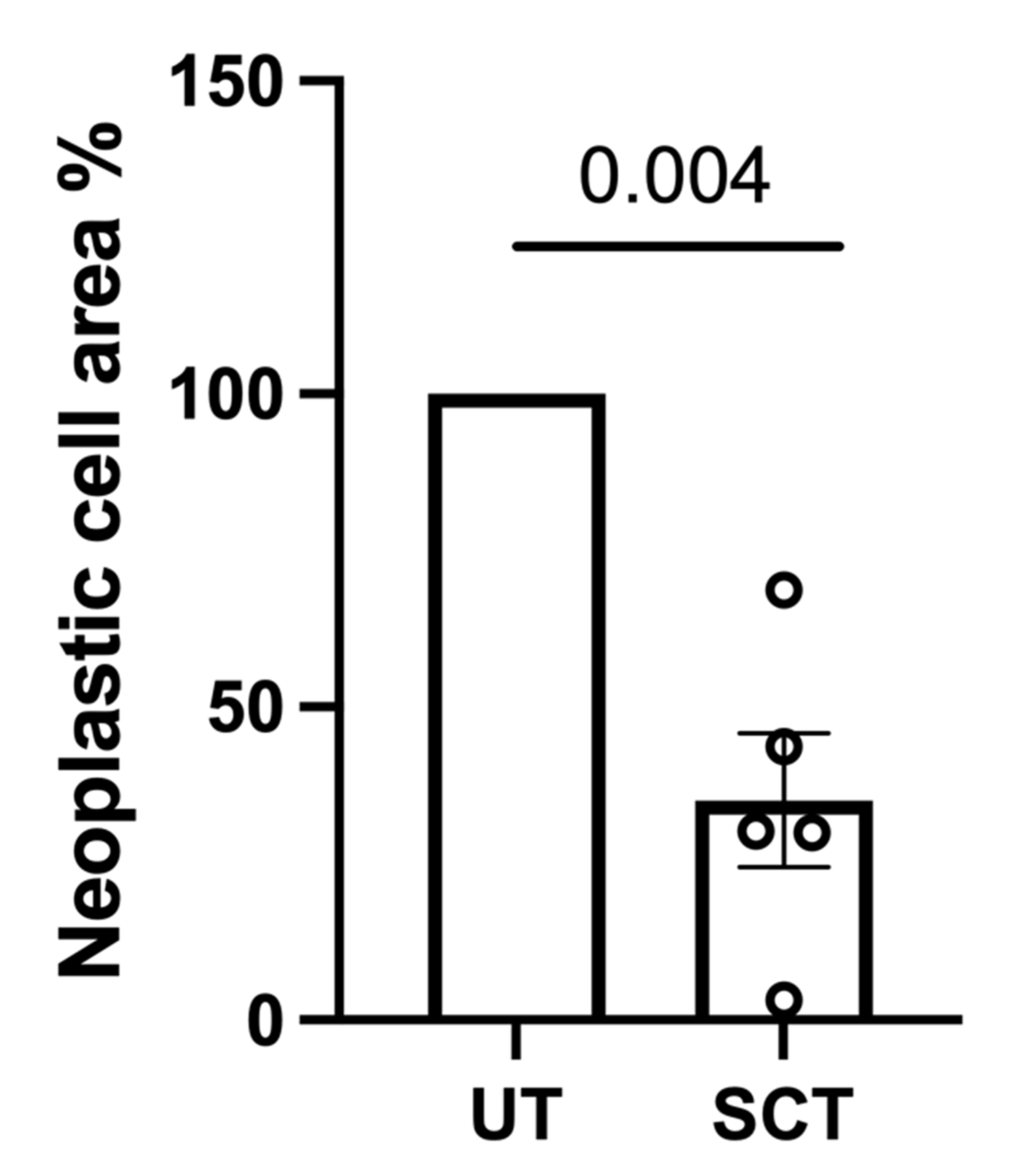


**Figure S9. Quantification of neoplastic cell area in ovarian cancer Slow-Frozen specimens after standard chemotherapy (SCT) treatment.** Quantification of the hematoxylin and eosin (HE) staining in HGSC SF specimens of untreated (UT) and SCT-treated samples cultured in U-CUP. Bar-plot indicates the percentage of neoplastic cell area with respect to the area of the specimen. Data relative to each specimen were normalized on the respective UT samples. QuPath was used for quantification. Mean value is shown (n=5), one sample t-test was applied for comparison and p-value is indicated for statistical comparison.
